# Modeling Techniques and Boundary Conditions in Abdominal Aortic Aneurysm Analysis: Latest Developments in Simulation and Integration of Machine Learning and Data-Driven Approaches

**DOI:** 10.3390/bioengineering12050437

**Published:** 2025-04-22

**Authors:** Burcu Ramazanli, Oyku Yagmur, Efe Cesur Sarioglu, Huseyin Enes Salman

**Affiliations:** 1 School of Information Technologies and Engineering, ADA University, Baku AZ1008, Azerbaijan; 2 Department of Mechanical Engineering, TOBB University of Economics and Technology, Ankara 06560, Türkiye; oyagmur@etu.edu.tr (O.Y.); efecesur.sarioglu@etu.edu.tr (E.C.S.); hsalman@etu.edu.tr (H.E.S.)

**Keywords:** abdominal aortic aneurysm, biomechanics, hemodynamics, fluid–structure interaction, boundary conditions, Windkessel model, machine learning, deep learning, data-driven techniques

## Abstract

Research on abdominal aortic aneurysms (AAAs) primarily focuses on developing a clear understanding of the initiation, progression, and treatment of AAA through improved model accuracy. High-fidelity hemodynamic and biomechanical predictions are essential for clinicians to optimize preoperative planning and minimize therapeutic risks. Computational fluid dynamics (CFDs), finite element analysis (FEA), and fluid-structure interaction (FSI) are widely used to simulate AAA hemodynamics and biomechanics. However, the accuracy of these simulations depends on the utilization of realistic and sophisticated boundary conditions (BCs), which are essential for properly integrating the AAA with the rest of the cardiovascular system. Recent advances in machine learning (ML) techniques have introduced faster, data-driven surrogates for AAA modeling. These approaches can accelerate segmentation, predict hemodynamics and biomechanics, and assess disease progression. However, their reliability depends on high-quality training data derived from CFDs and FEA simulations, where BC modeling plays a crucial role. Accurate BCs can enhance ML predictions, increasing the clinical applicability. This paper reviews existing BC models, discussing their limitations and technical challenges. Additionally, recent advancements in ML and data-driven techniques are explored, discussing their current states, future directions, common algorithms, and limitations.

## 1. Introduction

An abdominal aortic aneurysm (AAA) is formed when the wall of the abdominal aorta weakens, leading to a localized balloon-like structure. According to their position, AAAs can be classified as supraceliac, juxtarenal, infrarenal, and aortoiliac [1]. Infrarenal aneurysms are diagnosed when the diameter of the aorta exceeds 1.5 times its normal size, approximately reaching about 3 cm [2]. The risk of aneurysm rupture increases with the diameter. For aneurysms larger than 6 cm in diameter, the risk of rupture ranges between 10 and 20% per year [3]. However, the diameter cannot solely predict the rupture risk because clinical studies have documented ruptures in AAAs with diameters less than 5 cm [4]. AAAs develop often without showing any symptoms, which results in severe cases of AAA being undiagnosed [2].

The aorta wall has a thickness of around 1.5 mm, composed of three primary layers (intima, media, and adventitia), which include smooth muscle cells, collagen, and elastin fibers embedded in the ground matrix [5]. Their intrinsic mechanical properties enable the aorta to sustain loads at higher pressures. When pathophysiologically examined, an aneurysm can be described as an expansion of the vessel wall due to a thinning structure and the impact of mechanical stresses. Inflammation, oxidative stress, smooth-muscle-cell death, and matrix degradation are the main causes of thin vessel wall structures [6]. Moreover, hemodynamic factors, such as blood pressure, flow velocity, and resistance against flow, affect the arterial wall. A critical measure of this influence is the wall shear stress (WSS), which represents the frictional force exerted by blood flow along the artery wall. While the WSS acts tangentially in the direction of blood flow, blood pressure exerts a normal force toward the artery wall. These combined forces generate peripheral stresses, which cause vessels to stretch and deform. Furthermore, the areas of higher pressure indicate where the aneurysm may expand [7]. The relative wall strain (RWS) quantifies these dynamic effects caused by the peripheral stresses [3].

Blood flow decelerates when it crosses an aneurysm due to the expansion in the cross-sectional area, causing disturbed flow patterns and generating vortical structures. The innermost layer of the vessel wall (the intima layer) is a membrane covered with endothelial cells reacting to the disturbed flow and WSS variations by releasing inflammatory markers [5,8]. As a result, the low-velocity regions with vortices and WSS variations promote the accumulation of the platelets in that area. With the accumulation of endothelial cells, an intraluminal thrombus (ILT) may form which might lead to the further degradation of the wall [7].

There are many modeling and simulation studies that have been commonly used in recent years to investigate the hemodynamic and biomechanical properties of AAAs and their rupture risk. Rupture tends to occur in areas of low WSS and flow recirculation, rather than in regions of high pressure and WSS, which conflicts with earlier finite element analysis (FEA) studies [9]. The AAA wall stress distribution is significantly influenced by aneurysm asymmetry and wall thickness variations. Models with variable wall thickness have shown up to a four-fold increase in peak wall stress compared to those with uniform thickness. Accuracy of wall thickness in computational models to predict AAA rupture risk is emphasized by combining FEA and the fluid–structure interaction (FSI) [10]. Patient-specific FSI models of AAAs have proved that wall stress distribution and WSS are thoroughly affected by the interaction between blood flow and the arterial wall. These models show that high wall stress regions often align with complex flow patterns, which carry higher risk in the context of ruptures. Considering fluid dynamics together with wall interactions offers a more realistic simulation of in vivo conditions, compared to models that do not include the flow effects [11].

The accurate modeling of AAAs is clinically relevant due to its impact on predicting rupture risk and guiding surgical decisions. The integration of advanced modeling techniques into clinical practice helps avoid unnecessary surgical operations and improves risk assessment. Rigorous biomechanical analysis is important for the clinical detection of AAA rupture risk by using computational methods. FSI models that incorporate patient-specific geometries with accurate wall thickness and boundary conditions derived from advanced imaging techniques, such as phase-contrast MRI provide higher accuracy in predicting arterial wall stress and strain, which helps to recognize the smaller aneurysms with a high risk of rupture [12,13]. The integration of machine learning algorithms significantly improves the classification accuracy of symptomatic and asymptomatic AAAs. It is stated that the generalized additive model classifier achieved the highest accuracy using the combination of seven markers. Integrating patient-specific geometric and biomechanical properties may provide a more accurate rupture risk [14,15]. Integrating patient-specific data into these models will likely enhance the precision of AAA rupture predictions with the development of computational power and machine learning algorithms and this will improve clinical decision-making and patient outcomes.

In summary, the recent advancements in computational techniques have enabled a more comprehensive understanding of the biomechanical and hemodynamic parameters associated with AAA rupture risk. In computational models, the use of realistic and sophisticated BCs plays a pivotal role in capturing the realistic physiological environment within the AAA. Accurate representation of these BCs at the inlet and outlet of the fluid domain, along with precise modeling of wall mechanics, is essential for enhancing the reliability of simulations. Such developments not only refine predictions of arterial stress and strain but also support the integration of emerging technologies, such as machine learning algorithms, into AAA analysis. These advancements bridge the gap between theoretical models and their practical applications in clinical settings, promoting a more personalized approach to patient care.

This paper provides a comprehensive review of the diverse range of BCs employed in AAA simulations, focusing on their application at the inlet and outlet of the fluid domain, as well as wall conditions. Realistic BCs, such as the Womersley profile, Windkessel model, and fluid–structure interaction (FSI) techniques, are critical for accurate AAA simulations but are often absent in standard computational fluid dynamics (CFDs) solvers due to their technical complexity and the need for a detailed understanding of the underlying clinical issues. This review aims to address these challenges by exploring the physical and mathematical principles behind these BCs, offering valuable insights to researchers. Additionally, key findings from the literature are summarized, highlighting the limitations and technical barriers of the existing models. This paper concludes by discussing recent advancements in predicting hemodynamic and biomechanical parameters and disease progression through machine learning (ML) and data-driven techniques, with a focus on commonly used algorithms, their limitations, and potential future directions.

This paper provides a comprehensive review of the modeling techniques used in high-fidelity AAA hemodynamics and biomechanics, especially focusing on boundary conditions. While numerous reviews exist on this topic, the literature lacks a critical assessment comparing the different boundary condition models commonly used in studies. In this paper, we provide a detailed examination of the existing models, ranging from the simplest to the most sophisticated, by explaining their physical and mathematical background in detail. This gap is particularly significant for studies implementing ML models to predict AAA hemodynamics and biomechanics, as they rely on CFDs/FEA-generated datasets. The accuracy and reliability of ML-driven predictions depend on the fidelity of the BC models, which highlights the necessity of this critical review.

A structured literature search was conducted using databases such as PubMed, Web of Science, and Scopus, with keywords including ‘AAA biomechanics and hemodynamics’, ‘AAA hemodynamics with Windkessel’, ‘AAA hemodynamics with Womersley’, ‘FSI modeling of AAA’, ‘machine learning in AAA’, and ‘CFDs-based ML applications.’ Considering the rapid advancements in the field of artificial intelligence and its applications in medicine, AI-related papers were selected mostly from 2020 and later. Additionally, papers related to AAA modeling, diagnosis, and medical interpretations were selected mostly from 2017 and later in order to be up to date. In this study, SCIE-indexed articles were primarily used, and other resources were taken into consideration.

## 2. Computational Techniques for AAA Simulations

In the modeling of cardiovascular systems, various techniques have been developed. These can be categorized into low-dimensional (0D and 1D) [16,17,18,19] and high-dimensional (2D and 3D) [17,20,21,22,23] methods, based on the dimensionality of the simulation [18,24,25,26]. Low-dimensional techniques are computationally affordable and suitable for capturing general flow features across the entire cardiovascular system [27]. Specifically, 1D models solve the Navier–Stokes equations along the arterial tree to predict the flow profiles and pressure waves as they propagate throughout the system [17,22,27,28,29,30,31]. High-dimensional models can resolve detailed hemodynamic and biomechanical features in all spatial dimensions by solving governing equations in the solid and fluid domains. An AAA has a unique and complex geometry, with a wall thickness of around 1.5 mm [32]. Therefore, 3D patient-specific simulations are essential to achieve high-fidelity hemodynamic and biomechanic predictions.

### 2.1. Patient-Specific Modeling Approaches

Patient-specific modeling increases diagnostic and treatment accuracy by providing an examination adapted to individual anatomical differences. Non-invasive imaging methods, such as magnetic resonance imaging (MRI) [33,34,35,36], time-resolved three-dimensional ultrasound (3D+t US) [37,38], and computed tomography angiography (CTA), are typically used to extract patient-specific geometries in AAAs [39,40,41,42,43]. When the non-invasive methods are inadequate to determine the patient-specific geometry, invasive methods can be applied to provide more detailed information, as imaging is performed in a more limited area. For instance, intravascular ultrasound (IVUS) is performed by inserting a catheter containing an ultrasound probe into a vein, allowing the probe to capture images within the vessel. The use of IVUS, especially in cases of AAAs, eliminates the use of contrast agents used in CTA. Patients who underwent CTA during endovascular repair (EVAR) procedures have shown deteriorating renal function over time [44]. Angiography methods are also important tools that can be used to model the geometric structure. For example, 3D rotational angiography (3DRA) allows visualization of aneurysm structures of the order of 3 mm [45], and other traditional angiography methods can be applied for the aneurysms emerging in larger vessels, such as the aorta.

**Figure 1:**
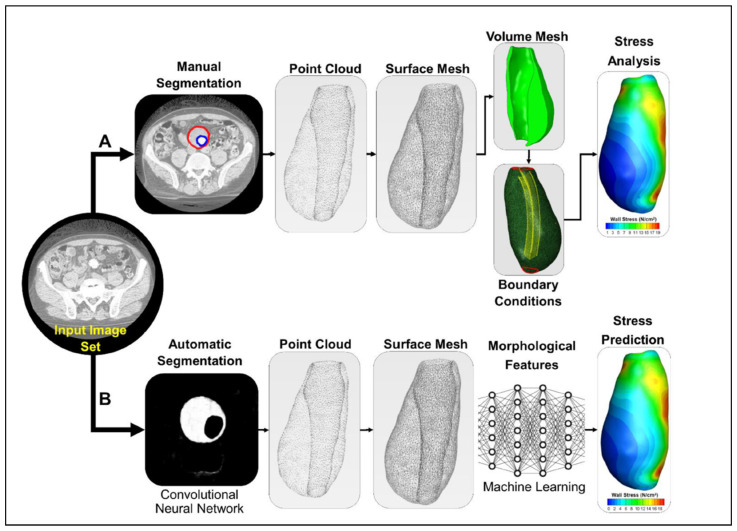
Workflow of the 3D AAA model for obtaining the CTA images of the abdominal aorta and segmentation of the vessel lumen manually, or using a deep-learning algorithm, generated 3D models, mesh generation, numerical simulation, and data analysis. Reproduced from [51] under the terms of the CC BY-NC-ND 4.0 license.

Following patient-specific medical image acquisition in the DICOM (Digital Imaging and Communications in Medicine) format, segmentation software, such as MIMICS 24.0 (Materialize, Leuven, Belgium), SimVascular 2.0, VMTK 1.4, and ITK-SNAP 42.2, reconstruct 3D models. Deep learning tools, such as U-Net 3D [46], SegNet [47], 3DResUNet [48], attention-based residual U-Net (ARU-Net) [49], and Context-Aware Cascaded U-Net (CACU-Net) [50] architectures offer significantly faster segmentation process compared to traditional algorithms. The overall workflow of segmenting is represented in Figure 1. The lumen and AAA walls are segmented separately to differentiate between these zones. The range of AAA thickness is within 0.23–4.26 mm, with a median wall thickness of 1.48 mm [32]. The distribution of wall thickness in AAAs is heterogeneous and changes from patient to patient. The geometrical structure of the wall has a significant importance in withstanding the dynamic loads, and the careful segmentation of the wall is an important step in computational modeling.

**Figure 2:**
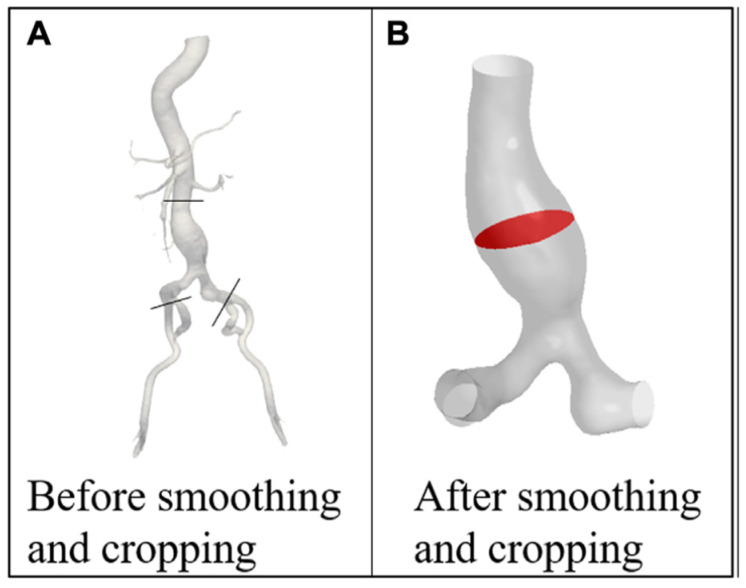
Models of the patient (**A**) before and (**B**) after smoothing and cropping. Reproduced from [53] under the terms of the CC BY 4.0 license.

**Figure 3:**
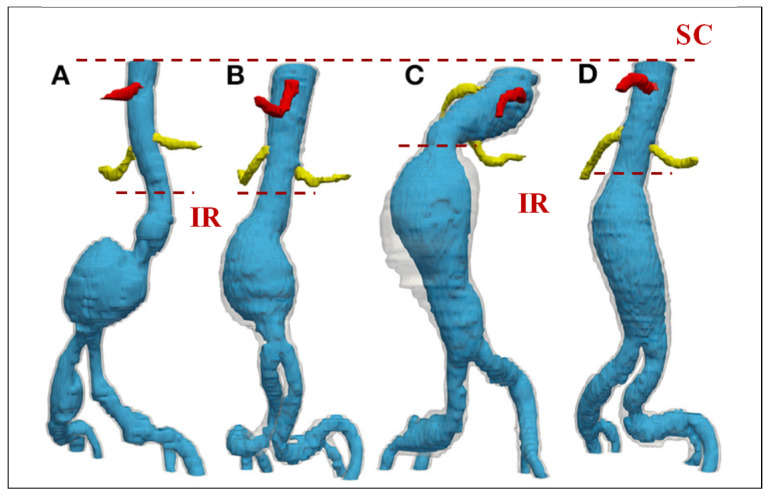
3D reconstructions obtained from the deep-learning-based segmentation results for four different patients (**A**–**D**). The lumen is in blue, the aortic ILT/wall is transparent, and the celiac and renal arteries are shown in red and yellow, respectively. Reproduced from [63] under the terms of the CC BY 4.0 license.

Extracting comprehensive CTA images of the entire vascular network can introduce additional challenges. Moreover, large-scale, image-based simulations of the entire arterial network are computationally demanding [16,52]. The common approach is to focus on a specific region of interest by cutting the aorta at certain boundaries. As illustrated in Figure 2, the region of interest is specified and truncated from the rest of the geometry by cutting the domain at the inlet and outlet boundaries during the segmentation process [5,13,53,54,55,56,57,58,59,60]. Several segmented 3D AAA models are presented in Figure 3. The supraceliac region (SC) marks the boundary between the descending and abdominal aorta. Downstream from the SC, the aorta branches into the celiac trunk (CT) and the superior mesenteric (SM), left renal (LR), right renal (RR), and accessory right renal (ARR) arteries. The aorta segment below the renal arteries is called the infrarenal region (IR), which bifurcates into the left central iliac (LCI) and right central iliac (RCI) arteries [35,61,62].

The placement of the inlet boundary varies depending on the hemodynamic parameters under investigation. Some studies position the inlet by dividing the region of interest from the aorta at the SC [62,64,65], while others cut the aorta from the IR [10,66,67], depending on the type of aneurysm. The number of outlet boundaries varies based on the position of the inlet. When the inlet is positioned in the SC, the model typically includes at least six outlets, corresponding to the CT, SM, LR, RR, LCI, and RCI arteries [35,61,62]. In contrast, placing the inlet in the IR reduces the number of outlets to at least two, located at the LCI and RCI arteries. However, the number of outlets may increase if additional arterial branches supplying blood to organs are considered. For example, the CT bifurcates into the hepatic (H) and splenic (S) arteries, while central iliac arteries further divide into the external iliac (LEI and REI) and internal iliac (LII and RII) arteries. Therefore, the total number of outlets may vary, depending on the complexity and specific requirements of the simulation. In the following section, numerical analysis is explained by providing the details of the governing equations, material properties, and boundary conditions used in the solid and fluid domains.

### 2.2. Analysis of the Fluid Domain

Computational fluid dynamics (CFDs) is a numerical technique used to determine the main hemodynamic parameters, such as pressure and velocity fields, in the overall fluid domain. In this technique, the following governing equations are numerically solved across a computational mesh to characterize the flow properties, as given in Equations (1) and (2).

(1) ∇.v=0

(2) $$\rho_{f}\frac{\partial v}{\partial t}+\rho_{f}\left(\left(v-w\right).\nabla \right)v=\nabla .\tau_{f}+f_{f}$$

Equations (1) and (2) are continuity and Navier–Stokes equations, respectively. The term denoted by $f_{f}$ is the external force acting per unit volume of fluid. The parameters of $v,\rho_{f}$ and $\tau_{f}$ define the velocity vector, density, and stress tensor of the fluid, respectively. The parameter of w denotes the moving coordinate velocity [67]. In the ALE formulation, v−w is denoted as the relative velocity of the fluid with respect to the moving coordinate velocity [68,69]. Both pressure and shear stresses are calculated for determining the stress tensors, as given in Equation (3).

(3) $$\tau_{f}=-pI+2\mu \left(\dot{\gamma }\right)\varepsilon$$

I defines the identity tensor, p denotes the pressure, and μ denotes the dynamic viscosity. The parameter of $\dot{\gamma }$ defines the shear rate, and ε denotes the strain rate tensor, given in Equation (4).

(4) $$\varepsilon =\frac{1}{2}\left(\nabla v+{\nabla v}^{T}\right)$$

#### Constitutive Models for Blood

As a first approximation, blood can be assumed as a Newtonian fluid, with a constant dynamic viscosity across all shear rates ($\mu \left(\dot{\gamma }\right)=3.5\;cP$), and a constant mass density ($\rho_{f}=1.05\;g/{cm}^{3}$) [7,39,66,70]. However, at low shear rates, particularly below 100 s^−1^, the non-Newtonian behavior of blood becomes more prominent [71]. At low shear rates, the red blood cells (RBCs) aggregate and form rouleaux, which are rod-shaped stacks of individual cells [71]. Rouleaux aggregation disperses as the shear rate increases, reducing the viscosity of blood. The resulting shear-thinning behavior caused by rouleaux disaggregation in blood plasma is the principal cause of the non-Newtonian behavior of blood. In numerical studies, the shear-thinning behavior of the working fluid, which is blood, can be modeled using several constitutive models. Viscosity functions with bounded and non-zero limiting values of viscosity can be written in the general form given in Equation (5).

(5) $$\mu \left(\dot{\gamma }\right)=\mu_{\infty }+\left(\mu_{0}-\mu_{\infty }\right)F\left(\dot{\gamma }\right)$$

In Equation (5), $\mu_{0}\;and\;\mu_{\infty }$ are the asymptotic viscosity values at zero and infinite shear rates, and $F\left(\dot{\gamma }\right)$ is a shear dependent function, satisfying the following natural limit conditions given in Equations (6) and (7).

(6) $$F\left(\dot{\gamma }\right)=\frac{\mu \left(\dot{\gamma }\right)-\mu_{\infty }}{\mu_{0}-\mu_{\infty }}$$

(7) $$\underset{\dot{\gamma }\to 0}{\lim}F\left(\dot{\gamma }\right)=1,\;\underset{\dot{\gamma }\to \infty }{\lim}F\left(\dot{\gamma }\right)=0$$

Different choices for the function $F\left(\dot{\gamma }\right)$ correspond to different constitutive models for blood, with material constants depending on factors such as temperature, hematocrit, and plasma. In the literature, eight non-Newtonian models are commonly used to represent the shear-thinning behavior of blood: Carreau, Carreau–Yasuda [72,73,74,75,76], Casson, Quemada [77], Power, Cross, Simplified Cross, and Modified Cross [76,78,79,80,81,82,83,84,85,86], as summarized in Table A1 in Appendix A.

On the other hand, the behavior of RBCs cannot be fully characterized by viscous phenomena because they can be regarded as fluid-filled elastic cells [87]. Therefore, blood exhibits a viscoelastic nature due to RBCs’ viscous shear-thinning and elastic properties [88]. In addition, increasing hematocrit, which is the volumetric ratio of RBCs in blood, causes increasing viscosity in the blood and makes non-Newtonian behavior of blood more significant. However, as the shear rate increases beyond the low shear rate region, the shear-thinning characteristics diminish, and blood exhibits Newtonian behavior [71].

To account for the elastic nature of blood, the viscoelastic Oldroyd-B model is frequently implemented [89,90,91]. In that model, the stress tensor in the linear momentum equation is decomposed into viscous and elastic parts as $\tau_{f}=\tau_{v}+\tau_{e}$ [71,91]. The viscous part of the Oldroyd-B model, $\tau_{v}=2\mu_{v}(\dot{\gamma })\left(\nabla v+{\nabla v}^{T}\right)$, can be Newtonian or shear-thinning [88]. The elastic part satisfies the following constitutive equation given in Equation (8).

(8) $$\tau_{e}+\lambda_{1}\left(\frac{\partial \tau_{e}}{\partial t}+v\cdot \nabla \tau_{e}-\tau_{e}\cdot \nabla v-{\nabla v}^{T}\cdot \tau_{e}\right)=\mu_{e}\left(\nabla v+{\nabla v}^{T}\right)$$

In Equation (8), $\mu_{e}$ denotes the elastic viscosity coefficient, and $\lambda_{1}$ denotes the relaxation time [85]. For the blood, $\mu_{e}$ is used as 0.0004 Pa·s and $\lambda_{1}$ is used as 0.06 s [85].

### 2.3. Analysis of Solid Domain

The finite element analysis (FEA) is a numerical method that allows the calculation of stresses and strains within a solid region to understand the mechanical behavior of the solid structure under various conditions. The governing equation provided in Equation (9) is numerically solved across a mesh created throughout the entire solid domain.

(9) $$\nabla .\tau_{s}+f_{s}=\rho_{s}\frac{\partial^{2}u}{\partial t^{2}}$$

In Equation (9), $\tau_{s}$ defines the solid stress tensor, $f_{s}$ defines the body forces per unit volume, and u defines the structural displacements. By writing the stress variable in terms of strains using Hooke’s law, the differential equation can be written in terms of displacements, as given in Equation (10).

(10) $$\tau_{s}=C\varepsilon$$

In Equation (10), the stiffness tensor is defined as C, the solid strains are defined as ε, and a linear correlation is defined between the stress and strain. However, in many cardiovascular modeling studies, the solid region to be investigated does not exhibit a linear elastic behavior [92]. Modeling the AAA tissue requires properly defining the mechanical properties of the wall and ILT. The constitutive models of the AAA wall are developed, calibrated, and validated through tissue characterization experiments [93,94,95,96]. To fit stress–strain behaviors, uniaxial tensile tests are conducted using tissue samples gathered during surgery [92]. AAA wall tissue exhibits complex material properties due to its layered, fiber-oriented structure, including hyperelasticity (nonlinear stress–strain behavior), viscoelasticity (time-varying response due to relaxation), and anisotropy (direction-dependent characteristics due to the distribution of collagen fibers) [15,97].

#### 2.3.1. Constitutive Models for AAA Wall

As a first approximation, the aneurysm wall can be modeled as a single layer, with linear elastic properties and isotropic medium characteristics, using an elastic modulus of 2.7 MPa, a Poisson’s ratio of 0.45, and a mass density of 2000 $kg/m^{3}$ [55,56,98,99,100]. The elastic modulus ratio for three-layered models of intima–media–adventitia is ~1:3:2 [101,102], with an elastic modulus of 3.2, 4, 8, and 1.6 MPa, respectively [103,104].

While linear elastic description can be applied to a healthy aorta or an aneurysm with low curvature [105], the effective characterization of nonlinear stress–strain properties of the AAA wall depend on the tissue’s hyperelastic nature [95]. Fitting the hyperelastic constitutive models to the experimental data enables us to determine the model coefficients. Although collagen fiber number and orientation influence mechanical behavior, modeling with the isotropic strain energy function is common [37,54,75,87,106]. To improve modeling accuracy, constitutive models, including the anisotropy, are available, capturing the directional dependency of collagen fibers in the strain energy function [93,94,95,96,105,107,108,109].

#### 2.3.2. Hyperelastic and Isotropic Models

##### Mooney–Rivlin Model

In the literature, two- [13,106,110,111] and three-parameter [104] Mooney–Rivlin models are commonly used to model the nonlinear stress–strain nature of AAA tissue and described in Equations (11)–(13), respectively. The strain energy per unit volume (W) is defined as

(11) $$W=C_{10}\left(I_{1}-3\right)+C_{20}{\left(I_{1}-3\right)}^{2}+\frac{{\left(J-1\right)}^{2}}{D}$$

(12) $$I_{1}=trC=\lambda_{1}^{2}+\lambda_{2}^{2}+\lambda_{3}^{2}$$

(13) $$J=\lambda_{1}\lambda_{2}\lambda_{3}$$

In Equation (11), $C_{10}$ and $C_{20}$ are the material constants obtained by fitting the experimental data. $I_{1}$ is the first Green’s strain invariant, C is the right Cauchy–Green strain tensor, and $\lambda_{1},\;\lambda_{2},\lambda_{3}$ are the principal strains that determine the relative changes in length along each principal direction. J is the ratio of the deformed elastic volume to the undeformed volume of the material. For incompressible materials, J=1 due to zero change in material volume, that vanishes the last term in Equation (11). D is the material incompressibility term.

Considering the population averages [97,112,113], material properties are determined as $C_{10}=17.4\;N/{cm}^{2}$, and $C_{20}=188.1\;N/{cm}^{2}$ for a single-layered wall structure. However, the material constants show certain variations from patient to patient. The minimum, average, and maximum values of $C_{10}$ were reported as $15.2,17.4\;\mathrm{and}\;21.9\;N/{cm}^{2}$, respectively, while the corresponding values for $C_{20}$ were 117.6, 188.1, and 355.7 $N/{cm}^{2}$ [54,114]. These studies recommend using population-averaged values in simulations, as variations in the Mooney–Rivlin material constants have been shown to have a negligible impact on peak wall stress and wall displacement [105].

##### Yeoh Model

The generalized Yeoh strain energy density function, W, is given in Equation (14).

(14) $$W=\sum_{i=1}^{N}C_{i0}{\left(I_{1}-3\right)}^{i}$$

In Equation (14), $I_{1}$ is the first invariant of the right Cauchy–Green strain, and $C_{i0}$ is the stress-like material parameters identified from the experimental data [77,115]. For the second order Yeoh model, W is defined as given in Equation (15).

(15) $$W=C_{10}\left(I_{1}-3\right)+C_{20}{\left(I_{1}-3\right)}^{2}$$

In Equation (15), $C_{10}$ is used as $17.4\;N/{cm}^{2}$, and $C_{20}$ is used as $188.1\;N/{cm}^{2}$ [92]. For the fifth order Yeoh model, the material values are calculated from planar biaxial experimental data as $C_{10}=0.5\;N/{cm}^{2}$, $C_{20}=C_{30}=0,$ $C_{40}=370\;N/{cm}^{2}$, and $C_{50}=1374\;N/{cm}^{2}$.

#### 2.3.3. Hyperelastic and Anisotropic Models

To account for the anisotropic nature of the AAA tissue, the Fung-type strain energy density function [116] is commonly used in the literature, as given in Equations (16)–(19).

(16) $$W=K\left(e^{Q}-1\right)$$

(17) $$Q=C_{11}E_{1}^{2}+C_{22}E_{2}^{2}+C_{12}E_{1}E_{2}$$

(18) $$E_{1}=\frac{1}{2}\left(\lambda_{1}^{2}-1\right)$$

(19) $$E_{2}=\frac{1}{2}\left(\lambda_{2}^{2}-1\right)$$

In Equations (16) and (17), K is a material constant related to the stiffness of the tissue, while $C_{11}$, $C_{22},\;C_{12}$ are the material constants depending on the specific tissue directions, such as circumferential, longitudinal, and radial, to capture anisotropy [105,117]. In Equations (18) and (19), $E_{1}$ and $E_{2}$ are the Green strains in circumferential and longitudinal directions, while $\lambda_{1}\;\mathrm{and}\;\lambda_{2}$ are the principal stretches that determine the relative changes in length along two primary axes. By fitting the material parameters to the experimental data, the effect of anisotropic fiber orientations can be modeled. In addition to the Fung-type [118], the models offered by Holzapfel [96], Holzapfel and Gasser [119], and Choi and Vito [120] are commonly applied in the literature. Most of the studies combine the isotropic and anisotropic strain energy density functions, as given in Equation (20) [92].

(20) $$W=W_{ISO}+W_{ANISO}$$

In Equation (20), $W_{ISO}$ describes the energy stored within the extracellular matrix, and $W_{ANISO}$ demonstrates the effect of the embedded collagen fibers.

#### 2.3.4. Constitutive Models for ILT

While ILT consists of two layers [121], with the inner layer forming about one-third and the outer layer about two-thirds of its thickness, modeling it as a single-layer elastic structure is common [54,106,122]. The elastic modulus of ILT is relatively lower than that of the wall. When ILT is initially formed, the elastic modulus is nearly equal to 50 kPa, while it may reach up to 200 kPa due to the increasing rigidity of the ILT structure over time [54]. The Poisson’s ratio and mass density of ILT are generally used as 0.45 and 1050 $kg/{cm}^{3}$, respectively. The tensile tests have shown that ILT tissue has an isotropic behavior [105]. To increase accuracy, nonlinear, hyperelastic, and isotropic constitutive models for ILT developed by Martino et al. [121] can be used, as given in Equations (21) and (22).

(21) $$W=C_{01}\left(I_{2}-3\right)+C_{02}{\left(I_{2}-3\right)}^{2}$$

(22) $$I_{2}=\frac{1}{2}\left[{\left(trC\right)}^{2}-\left(trC^{2}\right)\right]=\lambda_{1}^{2}\lambda_{2}^{2}+\lambda_{2}^{2}\lambda_{3}^{2}+\lambda_{3}^{2}\lambda_{1}^{2}$$

In Equations (21) and (22), $C_{01}$ and $C_{02}$ are the material parameters, and $I_{2}$ is the second invariant of the left Cauchy–Green stretch tensor. For the ILTs’ structure, $C_{01}=3.37$ and $2.23\;N/{cm}^{2}$ and $C_{02}=3.47$ and $2.24\;N/{cm}^{2}$ for inner and outer layers, respectively. By considering ILT as a single layer, the material constants can be taken as $C_{01}=2.80$ and $C_{02}=2.86\;N/{cm}^{2}$. Martino et al. [121] conducted a comparative study using various parameters and concluded that suggested mean parameters are sufficient, eliminating the need for patient-specific material constants.

### 2.4. Coupling of Solid and Fluid Domains: Fluid–Structure Interaction (FSI)

Modeling the AAA wall as rigid and stationary is common in the literature due to its simplicity. In fact, the vascular layers are viscoelastic and subjected to large deformations by the blood flow-generated loads. Conversely, the motion of hyperelastic walls alters the shape of the fluid domain and hemodynamics within AAAs, which necessitates a simultaneous blood flow and wall deformation treatment. Compared to flexible walls, rigid wall assumption obtains elevated WSS values up to 50% [123], underestimates the vortex development [107], and fails to predict pressure wave propagation [124,125]. Therefore, the mutual interaction between wall deformation and blood hemodynamics requires an FSI coupling at the aorta wall.

The FSI is a multiphysics technique [69,123,126] that couples the fluid domain to the solid domain at the wall interface by exchanging the instantaneous fluid velocity and wall deformation values between domains. The domains do not overlap, and the two domains are coupled at the luminal surface by satisfying a set of physical interface conditions to ensure the compatibility of kinematics and tractions. The solid wall domain and ILT structure are modeled using finite-element analysis (FEA) by discretizing the momentum conservation given in Equation (9), while the CFDs method is used to discretize the Navier–Stokes Equations given in Equation (2) that resolves blood flow patterns. The solid domain moves through space and deforms under hemodynamic loads, altering the shape of the fluid domain and necessitating the use of the Arbitrary Lagrangian–Eulerian (ALE) formulation. The ALE description of the fluid domain offers a moving coordinate system to simulate a non-stationary solution domain [127]. In the ALE method, the mesh nodes may be displaced or fixed as Lagrangian and Eulerian descriptions. With this freedom, large distortions within the fluid domain can be managed with more precision [127].

**Figure 4:**
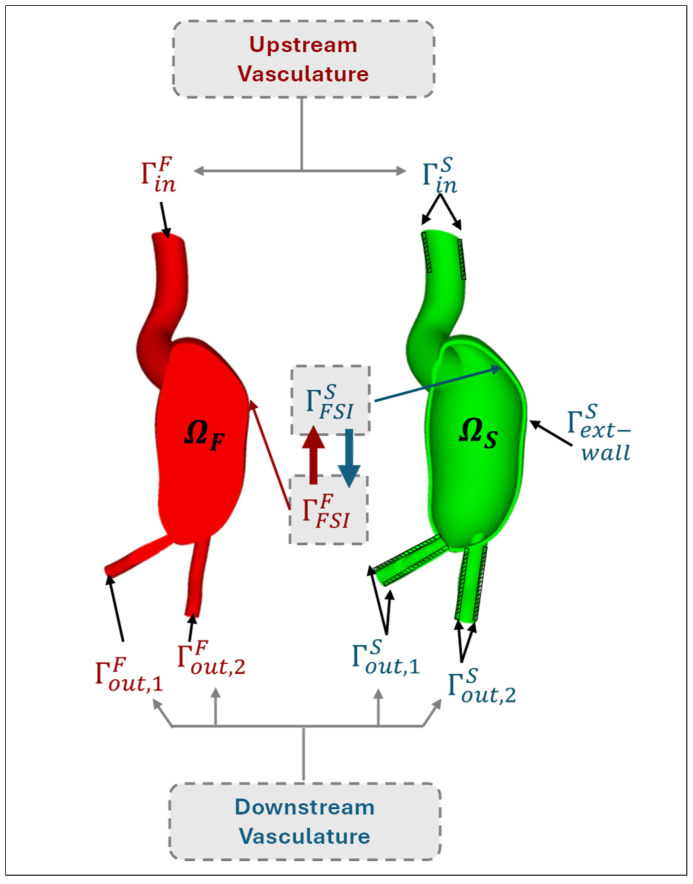
The fluid and solid domains of the AAA models with all the boundaries. Adapted from [54] under the terms of the CC BY 4.0 license.

Solving the governing equations within such truncated solid and fluid domains and coupling the solutions through the FSI procedure requires proper specification of the conditions at the boundaries of the domain, bringing forward concerns regarding the accuracy of the simulations. A high-fidelity, physiologically realistic modeling of AAA hemodynamics requires proper treatment of the conditions at the model boundaries. In Figure 4 and Table 1, boundaries are demonstrated for the fluid and solid domains. Appropriate specification of BCs at the inlet, outlets, and walls of the solid and fluid domains is essential to realistically couple velocity, pressure, stress distribution, kinematics, traction, and wave propagation in the upstream and downstream vasculature. The typical modeling techniques commonly employed in the literature for these boundaries are investigated in the following section.

## 3. Modeling Boundary Conditions in Fluid Domain

**Figure 5:**
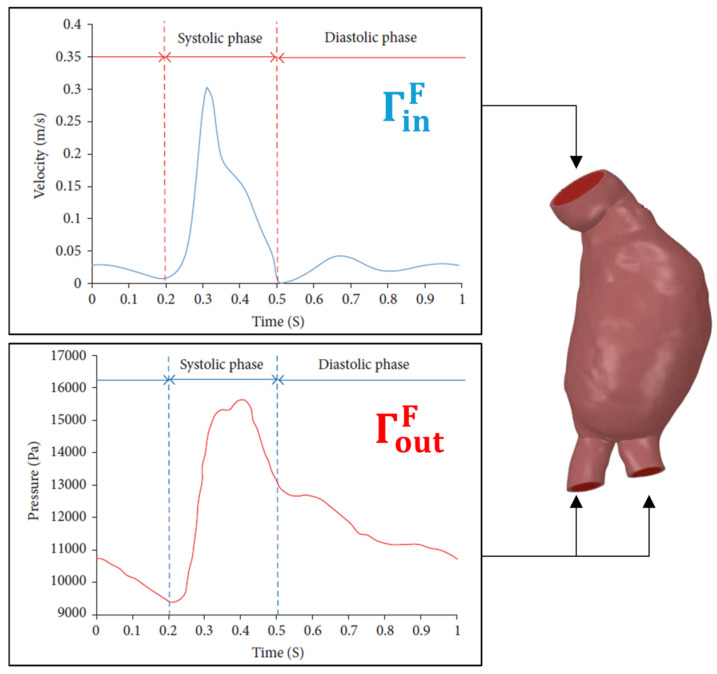
The time-varying inlet velocity (obtained from the flow rate) and outlet pressure profile. Adapted from [59,106] under the terms of the CC BY 4.0 license.

The accurate prediction of hemodynamic parameters, such as pressure and flow rate, requires proper coupling of the AAA domain with upstream and downstream hemodynamics at the truncated inlet and outlet boundaries. Proper coupling ensures that the simulated AAA hemodynamics interact with adjacent arterial compartments, closely reflecting physiological conditions. Typically, the time-varying inlet flow rate given in Figure 5 is prescribed for the inlet located at IR [54,66,107]. To enhance model accuracy, patient-specific inlet flow rates can be obtained through Doppler ultrasonography or phase-contrast magnetic resonance imaging (PC-MRI) [128]. At each point, spatial variations in flow velocity can be incorporated through customized velocity profiles based on the time-dependent flow rate. These profiles can be employed as idealized (i.e., flat, parabolic, or Womersley) or patient-specific. Patient-specific inlet velocity profiles can be measured non-invasively using 4D flow MRI [13,128,129], a three-dimensional, time-resolved type of PC-MRI that measures and visualizes the temporal evolution of blood flow within a specific 3D volume [76,79,129]. Alternatively, the inlet boundary can be connected to a lumped description of the heart [130,131,132]. However, using time-dependent pressure at the inlet is not a common approach due to the invasive nature and low precision of intraluminal pressure measurement, which requires catheter placement inside the inlet artery segment.

Applying outlet boundary conditions (BCs) is typically more complex. One challenge is the clinical measurement of intraluminal pressure, which requires catheter placement [13]. However, a significant portion of the cardiovascular system, including capillaries and veins, is located downstream of the aorta and substantially influences the AAA hemodynamics. Acquiring in vivo hemodynamic parameters at branching arteries using non-invasive techniques like magnetic resonance imaging (MRI) is challenging due to limitations in the spatial and temporal resolutions of the measuring devices and the physiological variations in patients during the imaging [133]. Van’t Veer et al. [134] compared non-invasive brachial cutoff blood pressure measurements with invasive catheter pressure measurements inside the AAA sac, reporting a 5% underestimation of systolic pressure and a 12% overestimation of diastolic pressure. The common approach, especially for geometries with one or two outlets, is to prescribe the time-varying pressure profile illustrated in Figure 5 at the outlets [32]. Various other techniques are available in the literature, including prescribed outlet pressure, flow-split method, lumped parameter models (e.g., 3-element Windkessel), and 1D distributed parameter techniques. The following section critically examines the common methods for defining boundary conditions, their applications, benefits, and drawbacks.

### 3.1. Inlet BCs

**Figure 6:**
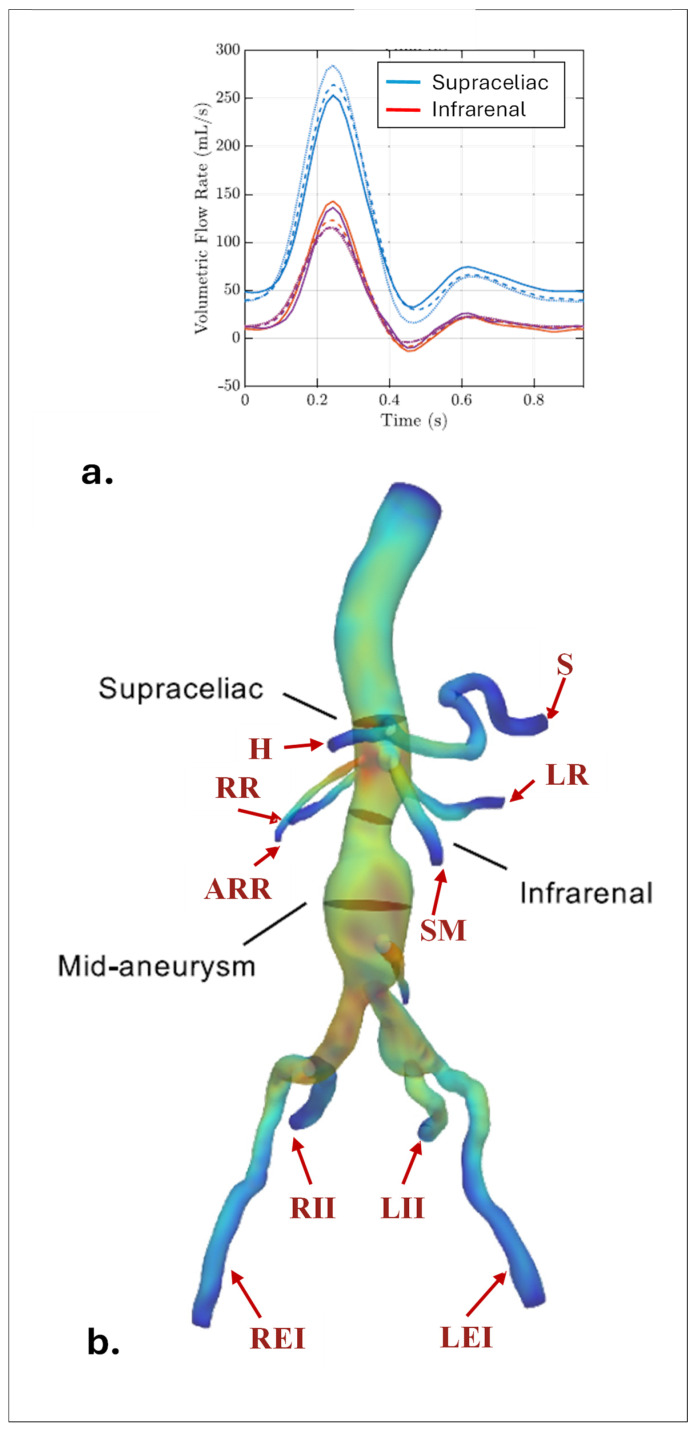
(**a**) The time-dependent flow rate at the supraceliac (SC) and at the infrarenal (IR) section. (**b**) The aorta branches at the downstream of SC: the celiac trunk (CT), superior mesenteric (SM), left renal (LR), right renal (RR), and accessory right renal (ARR) arteries. The aorta branches downstream of the IR: the right and left internal and external arteries (RII, REI, LII, and LEI). Adapted from [52] under the terms of the CC BY 4.0 license.

The time-varying flow rate at the inlet of an AAA can be integrated using customized velocity profiles, capturing spatial variations in flow velocity across the inlet section. The pattern of these flow rate waveforms is highly dependent on the inlet location. Depending on the requirements, the inlet can be positioned at the SC or IR locations. Figure 6 demonstrates the measured volumetric flow rates at the SC and IR regions [52]. Patient-specific profiles measured from the human aorta using the 4D flow PC-MRI technique, offer a more accurate representation of individual blood flow patterns, while idealized profiles (e.g., flat, parabolic, or Womersley) can illustrate general flow behaviors and are often used when patient data is unavailable [13,129,135].

The 4D flow PC-MRI images capture blood velocity vector fields in multiple sagittal slices of a healthy human aorta [129,136,137] at various time instants during the cardiac cycle. In this method, three-dimensional velocity maps at multiple time instants throughout the cardiac cycle are generated. The resulting pixel-based, time-varying velocity vectors are imposed on each voxel at the inlet section [137]. Chandra et al. [13] proposed a technique for mapping velocity vector field data onto the inlet boundary of a patient-specific AAA model, acquired on the IR plane. The inlet velocity profiles derived from 4D flow PC-MRI have a boundary that changes shape, size, and position due to aortic expansion and contraction during the systolic and diastolic phases. However, the cross-section of the inlet boundary in the CFDs models of AAA geometry remains fixed, both temporally and spatially [135]. To address this mismatch, Schwarz–Christoffel mapping aligns the datasets. Directly measuring in vivo inlet velocity profiles is still challenging because of the mismatch generated by such cardiac motion and the resolution of measuring devices [138,139]. Moreover, accessing complete high-quality, patient-specific geometry and inlet profile data is not always possible due to limited imaging facilities [139,140].

**Figure 7:**
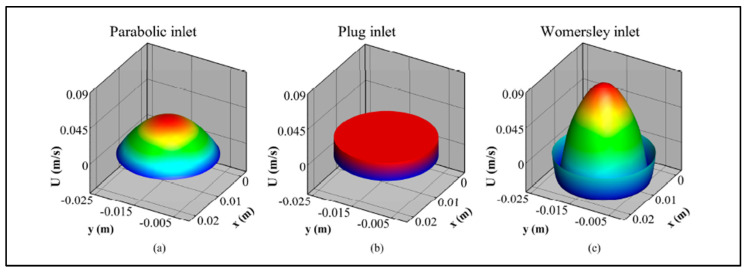
The idealized velocity profiles provided at the inlet of AAA models. (**a**) Parabolic; (**b**) plug (flat); and (**c**) Womersley profile. Reproduced from [63] under the terms of the CC BY 4.0 license.

Consequently, patient-specific velocity profiles are not commonly used as inlet BCs in AAA simulations. Instead, many studies employ artificial profiles, such as flat [56,141], parabolic [7,20,70,87,142,143], and Womersley [144,145,146,147]. Figure 7 shows the general pattern of the idealized velocity profiles supplied at the inlet of AAA models. Several studies have reported no significant difference between flow-MRI-derived profiles and artificial ones [148].

The artificial velocity profiles are calculated from time-dependent, patient-specific volumetric flow waveforms, represented in Figure 6, at the inlet section of the computational domain. Inlet flow rate waveforms are implemented to calculate the flat and parabolic velocity profiles using Equations (23) and (24).

(23) $$U_{flat}\left(t\right)=U\left(t\right)=\frac{2Q(t)}{\pi R^{2}}$$

(24) $$U_{par}\left(r,t\right)=\frac{2Q(t)}{\pi R^{2}}\left(1-{\left(\frac{r}{R}\right)}^{2}\right)$$

where $U_{flat}$ and $U_{par}$ are flat and parabolic velocities, Q(t) is the time-dependent flow rate, and R is the artery radius. As presented in Figure 7, the flat profile represents a uniform spatial distribution of the velocity vector at the inlet interface. The parabolic profile, derived from Poiseuille’s equation, exhibits a parabolic spatial distribution.

These profiles cannot fully capture all the transient effects introduced by the physiological flow rate waveforms, including flow reversal regions [149]. In 1955, Womersley developed an exact solution for incompressible, Newtonian fluid flow through a cylindrical, rigid blood vessel. This solution considers a pressure gradient that is a periodic function of time, which enables the capture of the reverse flow using the equations of motion and continuity. Figure 7 illustrates the velocity profile obtained by Womersley’s solution. To obtain the Womersley profile, it is necessary to write the time-dependent flow rate, Q(t)*,* in the harmonic form, as in Equation (25)

(25) $$Q\left(t\right)=\sum_{n=0}^{N}C_{n}e^{in\omega t}$$

In Equation (25), N is the total number of harmonic coefficients [21,148]. $C_{n}$’s are the Finite Fourier Transform (FFT) coefficients of that flow rate, while the term n=0 $\left(C_{0}\right)$ corresponds to a steady pressure gradient [148]. The Fourier series decomposition of the time-dependent flow rate should be performed to obtain the Fourier coefficients of the inlet flow rate waveform. The equation of the Womersley velocity profile is given in Equation (26), using the inlet flow rate waveform.

(26) $$\begin{array}{cc} U_{wom}\left(r,t\right)=\frac{2C_{0}}{\pi R^{2}} & \left(1-{\left(\frac{r}{R}\right)}^{2}\right)\\ & +\sum_{n=1}^{N}\frac{C_{n}}{\pi R^{2}\left(1-\frac{2J_{1}\left(i^{\frac{3}{2}}\alpha_{n}\right)}{i^{\frac{3}{2}}\alpha_{n}J_{0}\left(i^{\frac{3}{2}}\alpha_{n}\right)}\right)}\left[1-\frac{J_{0}\left(\alpha_{n}\frac{r}{R}i^{3/2}\right)}{J_{0}\left(\alpha_{n}i^{3/2}\right)}\right]e^{i\omega_{n}t} \end{array}$$

In Equation (26),$\;\alpha_{n}$ and $\omega_{n}$ are the $n^{th}$ term of the Womersley number and frequency, respectively. $J_{0}$ and $J_{1}$ are the Bessel function of the first kind of order zero and first, respectively [149]. In Supplementary Materials, the derivation of the Womersley profile and the MATLAB R2020b code is provided.

In Equation (26), the first term on the right-hand side equals the steady Poiseuille’s equation while the second term is the harmonic [149]. Although the Womersley profile presents transient effects of physiological flow, especially for large Womersley number (α) values, its application and implementation as an inlet boundary condition can be challenging due to the Bessel function and imaginary numbers that it contains [150,151]. Consequently, most studies in the literature utilize flat or parabolic profiles [152]. Current studies [21] show that the Womersley profile can be obtained by providing sufficient entrance length, at least $L_{ent}=3D$, and 10D is enough for parabolic and flat profiles, respectively.

Helical flow patterns in aortic hemodynamics have been observed in thoracic aorta studies [136,153]. Blood flow forms helical patterns in the ascending and descending aorta, as well as in the upstream sections of AAAs. This helical blood flow is a physiological characteristic where blood rotates and advances along the aortic axis [9,153]. These physiological helices potentially help maintain a WSS within the normal range [100,154], regulate flow, and protect vessels from thrombus deposition [155]. However, most AAA studies neglect the helical features of the incoming flow. To address this, Javadzadegan et al. [100,154] introduced a tangential velocity profile at the inlet to represent the helical pattern of the incoming flow, as presented in Equation (27).

(27) $$v_{\theta }=\omega r$$

(28) $$\omega =\frac{U}{R}C$$

In Equation (27), U is the streamwise velocity, $v_{\theta }$ is the local tangential velocity component, and r and R represent axial axis and undilated radius, respectively. ω is the spiral speed and C is a constant that controls the magnitude of the spiral speed. In the literature, C is typically defined as 1/6 for the aortic flows [156].

### 3.2. Outlet BCs

The purpose of the outlet BCs is to model the downstream vasculature that includes smaller arteries, arterioles, capillaries, venules, and veins that return blood to the heart [157]. Therefore, the choice of outlet BCs has a significant influence on velocity and pressure fields, and wave propagation linked to the wall properties in 3D AAA simulations [158]. Different outlet BCs have been adopted to better produce in vivo hemodynamic conditions in the cardiovascular system, including prescribed outlet pressure, the flow-split method [159], lumped parameter models, such as Windkessel, and resistance [23,24].

#### 3.2.1. Prescribed Outlet Pressure

Prescribing specific pressure at the outlets is common in AAA simulations. Prior studies typically implemented zero-gauge pressure at the outlets by setting $P_{out}=P_{atm}$ [160,161,162], and some current studies continue to use atmospheric pressure at the outlet for simplicity [145,147,163,164,165,166,167,168,169,170,171]. However, using zero-gauge pressure as an outlet boundary condition is insufficient for producing accurate flow and pressure features. This approach assumes that the outlet is open to the atmosphere, neglecting the effects of the posterior vasculature.

To avoid this unrealistic assumption, the common approach is prescribing a time-dependent pressure waveform instead [54,107]. However, invasively measuring pressure for branching arteries is challenging, so the general pressure waveform pattern demonstrated in Figure 5 is typically applied to all outlets. This method is frequently utilized in AAA simulations with an inlet at the IR region, where at most two outlets, the LCI and RCI, exist [7,53,72,74,108,172,173,174,175,176,177,178,179]. However, this method is less suitable for AAA simulations with an inlet at the SC. In that case, the increased number of outlets from branching arteries between the SC and IR segments brings complexities because the diameters, and the flow distribution among those arteries differs and the time-varying pressure data cannot be obtained simultaneously for each outlet. Reymond et al. [124,180] have obtained pressure waveforms for all outlet locations at the ascending aorta using a 1D model and validated with in vivo measurements to provide relevant physiological data. Furthermore, this method neglects the effect of arterial compliance, neglecting the downstream wave propagation. For a realistic simulation, matching the inlet flow waveform and outlet pressure distribution is essential and should be taken into consideration.

#### 3.2.2. Flow-Split Method

The flow-split method assigns specific flow rates to each outlet. These rates are determined through either formula-based calculations or direct in vivo measurements. Les et al. [35] reported the constant fractions of mean flows to each outlet. As shown in Figure 6a, the flow between the SC and IR regions, called upper branch vessel flows (UBVFs), is determined by subtracting the mean measured IR flow from the mean measured SC flow, corresponding to 1.31 and 3.51 L/min, respectively [32,35,61,62]. The remaining 2.2 L/min is distributed to upper vessel branches: 33% to the celiac trunk (CT), and 22.3% to superior mesenteric (SMA), the left renal (LR), and right renal (RR) arteries [35]. In the presence of accessory renal arteries, renal flow is divided proportional to the outlet area. The CT is then branched into hepatic (H) and splenic (S) arteries, with the flow distributed equally between them [35]. The remaining IR flow is divided equally into the two common iliac arteries, LCI and RCI, and 70% of this flow is diverted to external iliac arteries, while 30% is sent to internal iliac arteries [35,70,131,144]. These percentages are tabulated in Table 2.

To calculate the flow rate split among the outlets, Murray’s law is used [181,182]. The application of Murray’s law to estimate the flow splitting at artery bifurcations has been investigated in the literature. Murray [183] formulated that, in branching arteries, the flow in each outlet is proportional to the cross-sectional area of the bifurcated vessel. The general form of Murray’s law is given in Equation (29).

(29) $$\frac{Q^{i}}{Q_{tot}}=\frac{r_{i}^{n}}{\sum_{i=1}^{N_{outlet}}r_{i}^{n}}$$

In Equation (29), $Q^{i}$ is the flow rate at the $i^{th}$ outlet, $Q_{tot}$ is the total flow rate, $r_{i}$ is the radius of $i^{th}$ outlet, and $N_{outlet}$ is the number of outlets. The exponent n varies according to the arterial segment. Generally, it is considered to be 2 for the aortic segment [128,184]. Several studies have used an exponent n of 3 for AAAs [43,185,186,187]. However, specifying a fixed-flow rate at each outlet is not a realistic boundary condition because the flow division changes during the cardiac cycle, especially in deformable wall simulations where the outlet area changes continuously [188]. Furthermore, this BC neglects wave transmission to downstream vasculature [157].

#### 3.2.3. Lumped Parameter Model

To accurately model downstream vasculature at the outlets of the AAA, coupling the 3D computational domain with a reduced order (0D or 1D) is a common practice [133,189]. 0D models, also called lumped models, are governed by a group of ordinary differential equations (ODEs) that assume a spatially uniform distribution of blood pressure, P(t), and flow rate, $Q\left(t\right),$ within cardiovascular compartments at any time instant [158]. In vascular systems, the most widely used 0D models are Windkessel (WK) models [157,158]. This method represents downstream vasculature using lumped-parameter networks similar to electrical circuits consisting of capacitors, resistors, and inductors to relate $Q\left(t\right)$ and P(t) [184]. The elasticity of arterial walls allows them to expand and store large volumes of blood, then relax and push that blood downstream. This phenomenon, referred to as arterial compliance (C), is analogous to the behavior of a capacitor in an electrical circuit. Similarly, as blood flows downstream, the arteries bifurcate and form small-diameter capillaries and veins. The narrowing of arterial diameters generates significant peripheral resistance (R), analogous to electrical resistance [190]. In larger arteries, inertial energy is stored and released due to the acceleration or deceleration of fluid, which is expressed as an inertance (L), analogous to an electrical inductor. In this framework, blood flow and pressure are analogous to current and voltage, respectively. By applying Kirchoff’s voltage and current laws, ODEs governing the relationships between the P(t) and Q(t) are derived, called WK models [191,192].

**Figure 8:**
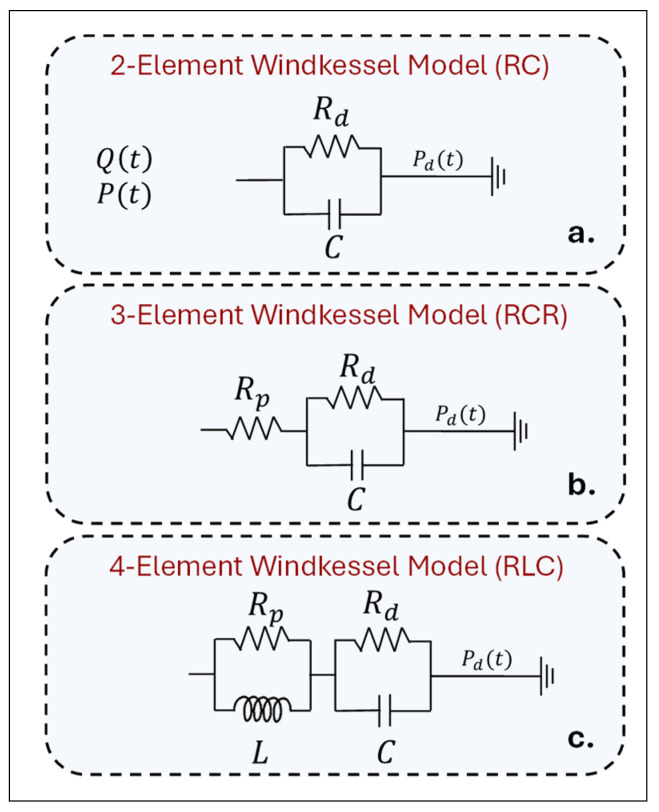
WK models: (**a**) 2-element (RC), (**b**) 3-element (RCR), and (**c**) 4-element (RLC).

WK models are classified based on the number of parameters or circuit elements (R, C, and L) [192]. Figure 8 illustrates the most common types: the two-element, three-element, and four-element WK models [191]. In the two-element WK model (WK2 or RC), P(t) is related to $Q\left(t\right)$ and the distal pressure, $P_{d}(t)$, through a parallel combination of distal resistance ($R_{d}$) and compliance (C). According to Poiseuille’s law, the resistance is inversely proportional to the fourth power in the artery radius, which makes the smallest arteries and arterioles the primary contributors to resistance in the cardiovascular system [192]. Consequently, the WK2 model accounts only for $R_{d}$, which represents the resistance of the downstream vasculature caused by the small arteries and the capillary bed [69,77,193]. However, the WK2 model has limitations in accurately describing the pressure–flow rate relationship because it omits the resistance of larger arteries [191,192]. To address this, three- and four-element WK (WK3 and WK4) models were developed [42,191,194]. The WK3 model sufficiently replicates the realistic downstream pressure at the outlet sections that are consistent with the experimental data. As a result, the WK3 model is commonly implemented in most hemodynamic simulations [140,152,191,195].

The WK3 model, often called the RCR model, consists of a proximal resistance ($R_{p}$) in series with a parallel arrangement of a distal resistance ($R_{d}$) and a compliance (C). $R_{p}$ describes the resistance in the large ascending aorta, proximal to the AAA. Based on the wave transmission theory [17,191], this helps absorb incoming pressure waves and reduce artificial wave reflections [133]. In the literature, the proximal and distal resistances are sometimes denoted as $R_{1}$ and $R_{2}$, respectively [131,133,196]. The sum of distal and proximal resistances is known as the total arterial resistance, $R_{T}=R_{p}+R_{d}$ [35,190]. The WK3 model describes the relationship between P(t) and $Q\left(t\right)$ using the ODE in Equation (30).

(30) $$P\left(t\right)=R_{T}Q\left(t\right)-R_{d}C\frac{dP\left(t\right)}{dt}+R_{p}R_{d}C\frac{dQ\left(t\right)}{dt}+P_{d}\left(t\right)+R_{d}C\frac{dP_{d}\left(t\right)}{dt}$$

In Equation (30), $P_{d}\left(t\right)$ represents the pressure at which blood flow to smaller arteries and capillaries in the vascular bed ceases [133,197], and is typically assumed to be zero in the literature [128,131,184]. For each outlet i, Equation (30) can be written as

(31) $$P^{i}\left(t\right)=R_{T}^{i}Q^{i}\left(t\right)-R_{d}^{i}C^{i}\frac{dP\left(t\right)}{dt}+R_{p}^{i}R_{d}^{i}C^{i}\frac{dQ^{i}\left(t\right)}{dt}\;\;\;\;\;\;i=1,2,3,\ldots ,N_{outlet}$$

**Figure 9:**
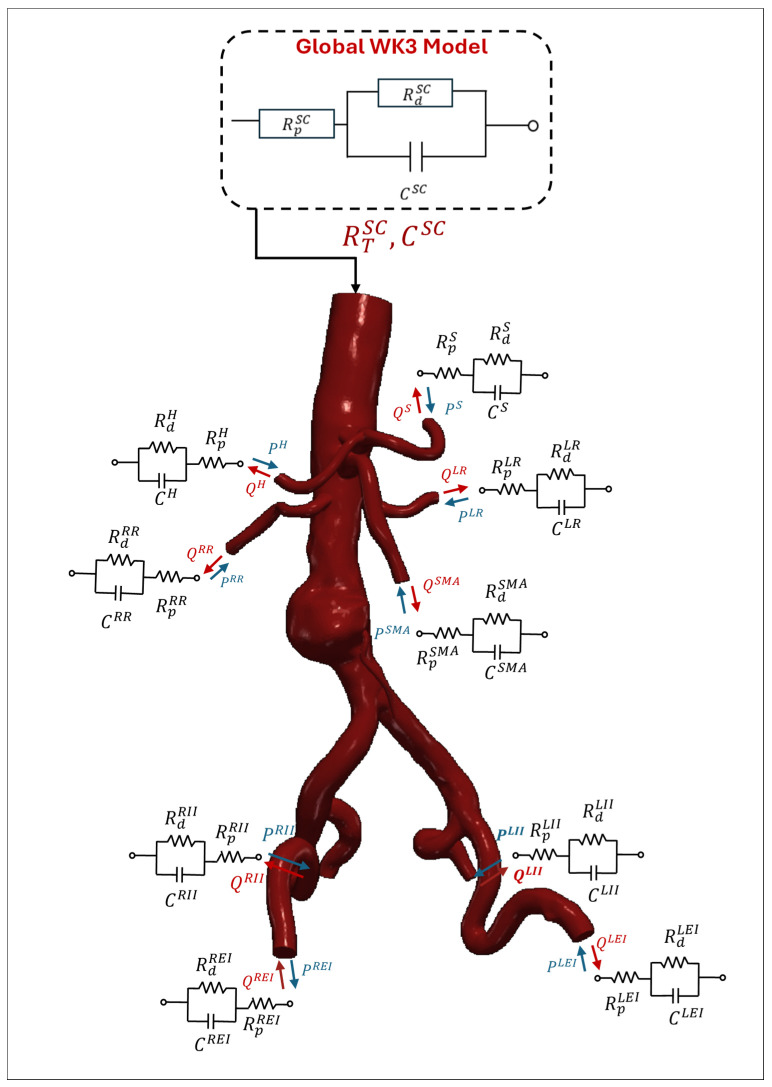
3D domain of AAAs and arterial branches coupled with separate 0D WK3 models at the outlets: S, H, SMA, LR, RR, LEI, LII, REI, and RII.

Figure 9 illustrates the 3D domain of AAAs and arterial branches coupled with separate WK3 models at the following outlets: S, H, SMA, LR, RR, LEI, LII, REI, and RII. This technique models downstream compartments in the arterial branch outlets in the 3D AAA domain. The parameters $(R_{T}^{i},\;R_{d}^{i},\;R_{p}^{i}$ and $C^{i})$ are constant for each outlet and independent of spatial dimensions [184]. By using $Q^{i}\left(t\right)$ calculated in the 3D simulation and estimating the WK3 parameters for each outlet i, $P^{i}\left(t\right)$ can be determined by solving Equation (31). Various techniques for estimating WK3 parameters exist in the literature. When patient-specific $P^{i}\left(t\right)$ and $Q^{i}\left(t\right)$ are available for each outlet, the least-square approach recently proposed by Romarowski et al. [198] is recommended. This method tunes the WK3 parameters to match measured in vivo patient-specific pressure and flow data [128]. However, both $P^{i}\left(t\right)$ and $Q^{i}\left(t\right)$ are not always simultaneously available, as most branches are quite narrow to make in vivo measurements.

Some studies have taken the parameters from the literature data to avoid the time-consuming parameter estimation process [152,199,200]. However, the parameters utilized should be carefully selected to match accurate patient pressure profiles because the values of WK3 parameters affect the hemodynamic parameters. In an uncertainty estimation study, Boccadifuoco et al. [184] reported that the $P\left(t\right)$ waveform at the aorta outlet is mainly affected by capacitance, C, while the $P_{max}$ is affected by $R_{p}$. Consequently, iterative solutions or approximations are frequently utilized to tune WK3 parameters [201]. Several studies implemented formulations of 1D modeling for parameter estimation [17,22,35,70,133,135,190,202]. A good agreement has been reported between the simulated pressure and flow rates and the in vivo measurements [35,133,157,158].

In this technique, a global WK3 model is created as the 0D representation of the 3D computational domain, as illustrated in Figure 9. The global WK3 parameters ($R_{T}^{SC},\;R_{d}^{SC},\;R_{p}^{SC}$ and $C^{SC})$ are defined at the inlet of the computational domain before calculating the local WK3 parameters $(R_{T}^{i},\;R_{d}^{i},\;R_{p}^{i}$ and $C^{i})$ for each outlet i. An initial estimate of the global compliance ($R_{T}^{SC})$ can be calculated by using Equations (32) and (33), defined for total resistance in 1D modeling [128,131,133]

(32) $$R_{T}^{SC}=\frac{P_{mean}^{SC}-P_{out}}{{\bar{Q}}^{SC}}$$

(33) $$P_{mean}^{SC}=\frac{1}{3}P_{sys}^{SC}+\frac{2}{3}P_{dia}^{SC}$$

In Equations (32) and (33), $P_{mean}^{SC},P_{sys}^{SC},\;\mathrm{and}\;P_{dia}^{SC}$ are the mean, systolic and diastolic pressures, and ${\bar{Q}}^{SC}$ is the time-averaged mean flow rate at the inlet [35,153,184,196,201]. The outlet pressure ($P_{out})$ is taken as 4.4 kPa in some studies [133], while generally is considered as $P_{out}=0$ [37,43]. In 1D simulations, the global compliance $\left(C^{SC}\right)$, which is the ratio of a volume change, ΔV, and the resulting pressure change, ΔP, is approximated as in Equation (34).

(34) $$C^{SC}=\frac{\Delta V}{\Delta P}=\frac{Q_{max}^{SC}-Q_{min}^{SC}}{P_{sys}^{SC}-P_{dia}^{SC}}\Delta t$$

In Equation (34), $Q_{max}^{SC}\;\mathrm{and}\;Q_{min}^{SC}$ are the maximum and minimum flow rates at the inlet, and Δt is the difference between the maximum and minimum flow rates [133,157,202]. Les et al. [35] determined the system’s global resistance and capacitance by iteratively solving for a pressure waveform instead of using Equations (32)–(34). This waveform was derived and further iterated as a function of the input SC flow waveform and the initial estimation of Windkessel model parameters.

The global parameters ($R_{T}^{SC}$ and $C^{SC}$) are used to calculate the local WK3 parameters at each outlet segment by utilizing Murray’s law [35,43,153,168,184,186]. Consequently, the total resistance and compliance at $i^{th}$ outlet can be calculated by using Equations (35) and (36), respectively.

(35) $$R_{T}^{i}=\frac{\sum_{i=1}^{N_{outlet}}A_{i}}{A_{i}}R_{T}=\frac{A_{tot}}{A_{i}}R_{T}^{SC}=\frac{{\bar{Q}}^{SC}}{Q^{i}}R_{T}^{SC}$$

(36) $$C^{i}=\frac{A_{i}}{\sum_{i=1}^{N_{outlet}}A_{i}}C^{SC}=\frac{A_{i}}{A_{tot}}C^{SC}=\frac{Q_{i}}{{\bar{Q}}^{SC}}C^{SC}$$

In Equations (35) and (36), $N_{outlet}$ is the number of outlets, and $A_{i}$ and $A_{tot}$ are the area of *ith* outlet and sum of the area of all the aortic outlets, respectively. Table 2 presents the ratios of time-averaged mean flow rates diverted to sub-branches in the AAA section after the SC region. These values, reported by Les et al. [35], are widely used in WK3 parameter tuning for AAA simulations. The ratio between the proximal and total resistance, $R_{p}^{i}/R_{T}^{i}$, is taken as 5.6% for most artery segments, with the exception of renal arteries [5,17,35,37,43,131,190,203,204]. Renal arteries, connected to kidneys, have relatively low $R_{d}^{i}$ at the vascular beds, resulting in an $R_{p}^{i}/R_{T}^{i}$ ratio of 28% [131]. Once the total resistance at the $i^{th}$ outlet is determined, the proximal and distal resistances can be calculated as

(37) $$R_{T}^{i}=R_{p}^{i}+R_{d}^{i};\;\;\;\;R_{p}^{i}=0.056R_{T}^{i};\;\;\;\;R_{d}^{i}=0.944R_{T}^{i}$$

**Figure 10:**
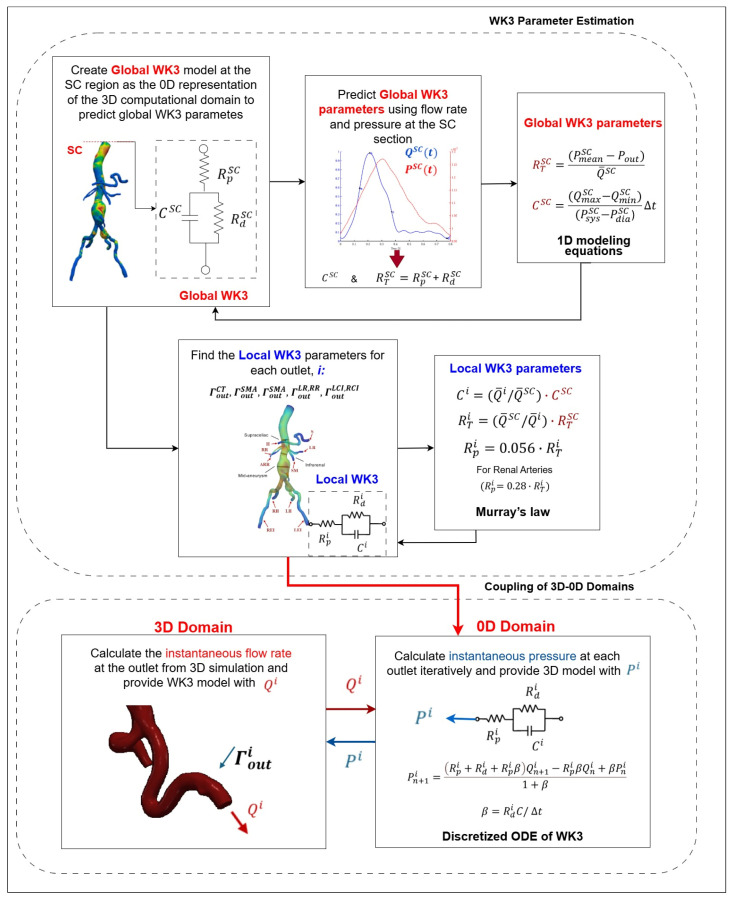
The flowchart of the global and local WK3 parameter estimation (using 1D equations and Murray’s Law) and the coupling of 3D-0D domains. Adapted from [52] under the terms of the CC BY 4.0 license.

The calculation of the local from the global WK3 parameters for the downstream CT, SMA, LR, RR, LCI, and RCI compartments is summarized in Table A2 in Appendix B. The flowchart in Figure 10 summarizes the overall process. Although these 1D formulations are frequently used in AAA simulations, concerns still exist regarding their applicability to detailed geometrical shapes and FSI simulations where the flow rate directed to each branch is not constant due to the arterial deformations [153].

To improve parameter estimation accuracy, various tuning methods have been developed, primarily for ascending aorta and aortic dissections. Jiang et al. [205,206] initially performed a CFDs simulation with zero-gauge pressure imposed at all outlets, calculating flow rates and pressures to tune the WK3 parameters. In subsequent rounds, they imposed the WK3 with estimated values, continuing until the pressure difference was less than 5%. Several studies estimated local parameters through iterative ODE solutions to match the physiological pressure distribution within specified systolic and diastolic limits for the AAA [36,37,203,207]. Some of the studies used general values like 120/80 mmHg [36,69,153,184,208,209]. The local WK3 parameters are adjusted until the desired waveform is achieved. Fonken et al. [37,204] measured patient-specific brachial blood pressure in a supine position using a brachial cuff. Alimohammadi et al. [196] measured minimum and maximum pressures at all branches of type-B aortic dissection, using a transfemoral flush angiographic catheter connected to a pressure transducer. To further improve estimation accuracy, Alimohammadi et al. [196] and Pant et al. [210] applied the data assimilation technique and unscented Kalman filters, respectively.

Spilker et al. [211] and Bonfanti et al. [131] used reduced model tuning techniques to enhance initial guess accuracy, and various CFDs simulation results have been used for further iterations to optimize local parameter fine-tuning. Li et al. [153] proposed a fast-estimating approach that eliminates the need for CFDs simulation iterations. They initially obtained total resistances as the input for each interface using 1D formulations, with systolic and diastolic pressures as 120/80 mmHg. The local WK3 parameters are then optimized using the pattern search algorithm from MATLAB’s global optimization toolbox.

After estimating the parameters, the coupling of 3D with the 0D domain is commonly done explicitly [212,213] or semi-implicitly. The derivative terms in Equation (31) are typically discretized, as in Equation (38), by using the backward Euler method [196].

(38) $$P_{n+1}^{i}=\frac{\left(R_{p}^{i}+R_{d}^{i}+R_{p}^{i}\beta \right)Q_{n+1}^{i}-R_{p}^{i}\beta Q_{n}^{i}+\beta P_{n}^{i}}{1+\beta }$$

In Equation (38), $\beta =R_{d}^{i}C/\;\Delta t$ [42]. To achieve appropriate coupling, the flow rate equality condition for the multiscale model at the interface, $Q_{3D}^{i}=Q_{0D}^{i}$, must be satisfied [196]. The instantaneous pressure, $P_{0D}^{i}(t)$, is calculated from Equation (38) by using the flow rate for the current solver loop, and $Q_{3D}^{i}(t)$ and the pressure and flow rate from the previous time step, $P_{0D}^{i}(t-\Delta t)$ and $Q_{3D}^{i}(t-\Delta t)$, respectively [196,213]. The resulting pressure, $P_{0D}^{i}\left(t\right),$ is then supplied back to the 3D domain as the uniform pressure BC at the current time step. For implicit coupling, $P_{0D}^{i}(t)$ and $Q_{3D}^{i}(t)$ are solved simultaneously in an iteration loop [214]. In explicit coupling, the pressure is calculated at the beginning of an iteration by using the flow rate value from the previous time step.

WK3 coupling isn’t available on every solver. SimVascular offers coupled WK2 and WK3 [195], while it is not defined in other software. To make such a multidomain solution in OpenFOAM, heamofoam [215] can be used. To use the WK3 BC in ANSYS (version 2025), a user-defined function (UDF) should be created to couple two domains. For CFX, the Fortran subroutine can be defined [196,208]. In some studies, 3D-0D coupling is not performed. In several studies [40,216,217], a time-dependent pressure waveform has been calculated through the WK3 and applied as the prescribed pressure BC at the outlets.

#### 3.2.4. Resistance BC

To relate the flow rate and the pressure at the outlets of the computational domain, some studies prefer to use resistance BC due to its simplicity [23,76,185,218]. In this technique, the resistive property of the downstream vasculature is imposed by a resistance parameter, while the compliance of the arteries is neglected using Equation (39).

(39) $$P_{out}^{i}\left(t\right)=P_{ref}+R_{out}^{i}Q_{out}^{i}\left(t\right)\;\;\;\;\;\;\;\;i=1,2,3,\ldots ,n_{outlet}$$

In Equation (39), $R_{out}^{i},$ $P_{out}^{i}(t)$, and $Q_{out}^{i}\left(t\right)$ are the resistance, time-dependent pressure, and flow rate at the $i^{th}$ outlet, and $P_{ref}$ is the reference pressure related to the venous pressure. $R_{out}^{i}$ is calculated by distributing the global resistance calculated from Equation (32) into branch arteries, using Equation (35). However, resistance BC is not the commonly preferred BC in AAA simulations since it severely impacts wave propagation phenomena [157,219].

## 4. Modeling Boundary Conditions in Solid Domain

### 4.1. Inlet and Outlet of the Wall

The branch arteries of the aorta produce a tethering effect on the AAA. To model this effect, zero rotation, and translation conditions are imposed at the inlet and outlet of the wall domain [10]. To further improve the accuracy, Scotti et al. [67] specified a 5% axial stretch on these boundaries [220] since the artery walls are physiologically under tension.

### 4.2. External Wall Boundary

The intra-abdominal pressure imposed by surrounding tissues and organs on the outer surface of the AAA wall requires consideration [221]. Most studies, however, assume a free stress condition, with zero pressure on the external AAA wall. This boundary condition can lead to non-physiological wall motion patterns [188], causing several researchers to use a constant intra-abdominal pressure of 12 mmHg [67]. Crosetto et al. [125] introduced a Robin condition which requires appropriate model parameters through curve fitting to the empirical data. Similarly, Moireau et al. [222,223] developed a BC along the outer wall of the thoracic aorta (TA), incorporating a viscoelastic term to account for surrounding tissue and organ support.

### 4.3. FSI Boundary: Coupling Solid and Fluid Domains

FSI modeling can be performed using several numerical approaches: one-way uncoupled [13], one-way coupled [64,224], and two-way coupled, both explicit and implicit [111]. In the one-way uncoupled method, the intraluminal pressure load is supplied to the FSI interface, and only solid mechanics equations are solved in the wall domain, without feeding the fluid domain with the pressures obtained by the solid domain. In two-way coupled methods, solid and fluid domains are simulated simultaneously, interacting through the FSI interface. Comparative studies [224] showed that the one-way FSI technique overestimates pressure, WSS, and strain in the fluid domain, while underestimating the von Mises stress and displacement. As shown in Equations (40)–(42), three conditions must be met at the FSI boundaries: the solid and fluid domain displacements must be compatible, boundary tractions must be in equilibrium, and fluid must follow the no-slip condition on the FSI boundary surface.

(40) $$d_{s}=d_{f}$$

(41) $$\tau_{s}\cdot {\hat{n}}_{s}=\tau_{f}\cdot {\hat{n}}_{f}$$

(42) $$v=\dot{d_{f}}$$

In Equations (40)–(42), $d,\tau ,\hat{n}$, and $d_{f}$ are the displacement vectors, stress tensors, and boundary normal vectors at the FSI boundary for the solid, *s*, and fluid, *f*, domains. In two-way explicit coupling, information exchange among the fluid and solid domains occurs explicitly, without iterations within each time step. To obtain converged results, small steps are required. In two-way implicit coupling, the fluid and solid equations are solved simultaneously at each time step, iteratively exchanging information until convergence. While this method requires high computational memory and excessive computational time, it is necessary to reach convergence for improving accuracy.

## 5. Important Post-Processing Indices

Hemodynamics inside an aneurysm sac can be quantified by several wall shear stress (WSS) descriptors, such as the time-averaged wall shear stress (TAWSS), oscillatory shear index (OSI), endothelial cell activation potential (ECAP), and relative residence time (RRT). Mathematical definitions of these descriptors are given in Equations (43)–(46) [225,226].

(43) $$TAWSS=\frac{1}{T}\int_{0}^{T}\left|\tau_{w}\right|dt$$

(44) $$OSI=0.5\left(1-\frac{\left|\frac{1}{T}\int_{0}^{T}\tau_{w}dt\right|}{\frac{1}{T}\int_{0}^{T}\left|\tau_{w}\right|dt}\right)$$

(45) $$ECAP=\frac{OSI}{TAWSS}$$

(46) $$RRT=\frac{1}{(1-2\cdot OSI)\cdot TAWSS}$$

where T and $\tau_{w}$ are the cardiac cycle period and the wall shear stress, respectively.

Several studies also reported a correlation between recirculation zones and WSS descriptors. The utilization of vortex fields may afford a more comprehensive understanding on ILT development and rupture mechanism, compared to the WSS descriptors [227,228]. In the literature, vortex structures are commonly quantified by Q, Δ, $\lambda_{2}$, $\lambda_{ci}$ (swirling strength) criteria [229,230] and rortex [231]. The equations for vortex identification criteria are given in Appendix C.

For the AAA wall, principal wall stresses, von Mises stress, and wall displacements are critical parameters. The von Mises stress, based on three principal stresses, is a measure of failure prediction, as given in Equation (47).

(47) $$\frac{1}{2}\left[{\left(\sigma_{1}-\sigma_{2}\right)}^{2}+{\left(\sigma_{2}-\sigma_{3}\right)}^{2}+{\left(\sigma_{3}-\sigma_{1}\right)}^{2}\right]>\sigma_{y}^{2}$$

In Equation (47), $\sigma_{1},\;\sigma_{2},\;\sigma_{3}$ are the principal stresses, and $\sigma_{y}$ is the uniaxial failure strength of the wall [32]. The term on the left-hand side is the square of the von Mises stress. Peak wall stress (PWS) is a critical indicator of rupture, occurring when the AAA wall strength is insufficient to withstand the PWS [232].

## 6. Recent Findings

To accurately simulate the AAA hemodynamics and wall mechanics, researchers have developed and implemented different techniques over a quarter century. Starting from the axisymmetric geometries supplied with simplified boundary conditions and material properties, in silico investigation of the AAA development and rupture characteristics has reached patient-specific geometries with realistic boundary conditions, enhancing the reliability of predictions. Intense research has accomplished coupling different computational domains solved by various methods, such as CFDs and FEA, at the wall interface by implementing the FSI technique, and different dimensions, such as 0D and 3D. The increasing use of medical imaging techniques, such as CT, MRI, and 3D ultrasound, enables the extraction of patient-specific geometries with accurate wall thickness distribution and with eliminated prestress that ensures precisely capturing the anatomical features of AAAs. The utilization of the 4D flow MRI and angiographic catheters provide time-dependent, patient-specific inlet velocity profiles and outlet pressure waveforms, respectively. With the help of biaxial and uniaxial tissue tests, accurate constitutive models with patient-specific material constants have been developed.

The utilization of 4D flow MRI inlet velocity profiles is common in thoracic aorta (TA) studies [79,128,136,140,207], while the implementation of artificial velocity profiles developed from patient-specific inflow waveforms at the SC or IR regions is standard practice in AAA simulations. Chandra et al. [13] obtained patient-specific velocity profiles at the IR region using the 4D flow PC-MRI technique and compared the results with simulations using artificial profiles. Their results showed that WSS, PWS, and strain are influenced by the hemodynamics from the applied inflow boundary condition. A comprehensive biomechanical approach for assessing AAA rupture risk should consider the interaction between the aortic wall and hemodynamics with patient-specific inflow boundary conditions [79,146]. Wei et al. [148] found no significant difference between PC-MRI measured and artificial inlet profiles, though the flat profile showed notable differences in Fontan hemodynamics. Ramazanli et al. [21] compared artificial velocity profiles for AAA hemodynamics and recommended the parabolic velocity profile for its simplicity. However, the flat profile is not recommended by many studies [5].

Prescribing pressure waveforms at the outlet of branching arteries remains common in AAA simulations, particularly for 3D domains starting after the IR section with two outlets. Wang et al. [5] compared various inlet and outlet BCs in an AAA domain with two outlets, using a two-way FSI model with hyperelastic and anisotropic properties. They prescribed parabolic and flat profiles at the inlet, along with pressure waveforms from the literature and the WK3 BCs at the outlet. Their comparison of average anTAWSS along AAAs, PWS, and maximum displacement showed that WK3 significantly affected all three measurements. The prescribed pressure BC overestimated the PWS and displacement while underestimating TAWSS.

Pirola et al. [128] applied a different approach, using patient-specific pressure waveforms measured at each thoracic aorta outlet before MRI geometry extraction. They compared a patient-specific outlet pressure BC with WK3 and zero pressure BCs, finding that outlet BC choice significantly impacts aorta hemodynamics. Their results showed that WK3 can accurately reproduce physiological aortic pressure waveforms, suggesting the use of the WK3 model when patient-specific pressures are unavailable. Madhavan and Kemmerling’s [152] comparison of WK2, WK3, and flow-splitting BCs revealed minimal variations between WK2 and WK3, though they found an 18% difference in TAWSS between the WK models and flow splitting. Boccadifuoco et al. [184] studied uncertainties in the WK3 parameters for both rigid and deformable wall models. Their stochastic analysis showed that compliance (C) has the greatest impact on hemodynamic predictions, compared to distal $\left(R_{d}\right)$ and proximal $\left(R_{p}\right)$ resistances. However, they concluded that the WK3 parameters may not be a major uncertainty source, particularly when considering wall elasticity.

In the literature, the rigid wall assumption is typically applied in a hemodynamic investigation of AAAs, resulting in underestimated PWS and overestimated velocity, WSS, and OSI values. Integrating FSI at the AAA wall interface increases the simulation accuracy by accounting for wall deformation. To further enhance reliability, researchers have developed constitutive models with patient-specific material constants for the wall layers and ILT structure. Comparative studies of elastic and hyperelastic models show that elastic models underestimate the von Mises stress on the AAA wall [75,104,233] and predict wall displacement about 20% greater than hyperelastic material [104]. Xenos et al. [122] compared the isotropic Mooney–Rivlin and anisotropic Holzapfel models, finding that the isotropic model underestimates the PWS. Similarly, Rissland et al. [234] discovered that isotropic models underestimate the wall deformation. Balzani et al. [109] compared the Neo-Hooken isotropic model with anisotropic models, concluding that isotropic models oversimplify arterial mechanical behavior. While anisotropic models are preferred, they significantly increase numerical complexity. To address this, Wang et al. [235] developed a combined strain energy function that models extracellular matrix energy as isotropic and collagen fiber effects as anisotropic.

Though AAA walls vary in thickness, most studies assume constant thickness due to geometry extraction challenges [106,236]. However, several studies show that wall thickness significantly affects wall biomechanical properties [99,237]. Raghavan et al. [238] observed that the minimum wall thickness could reach 0.23 mm around the rupture site. Scotti et al. [10] observed that varying wall thickness leads to an increase of the von Mises stress up to 4 times compared to the uniform thickness. Raut et al. [239] compared maximum principal stress and strain, strain energy density, and displacement magnitude across different thickness models. Their findings strongly support using patient-specific, regionally varying wall thickness from CT scan segmentation, particularly for FEA analysis of AAAs.

Addressing pre-stress in CT-reconstructed geometry improves the accuracy of the results. The reconstructed AAA geometry reflects a pressurized aortic state due to the physiological pressure during image acquisition, potentially leading to WSS distribution underestimation. Fonken et al. [37] implemented a backward incremental method (BIM) to estimate pre-stress in measured geometry. Omitting pre-stress leads to increased systolic displacements and decreased systolic wall stresses up to 77.8% and 54.2%, respectively.

In the literature, a vast number of studies have postulated a correlation between the wall shear stress parameters and intraluminal thrombus (ILT) formation [9,35,144,213]. The most well-known approach is that the low and oscillatory WSS may stimulate the endothelial cells and promote the inflammatory process, causing wall–cell adhesion due to platelet accumulation, forming ILT. WSS values lower than 1 Pa are considered as low WSS [39,171]. Most researchers agree that the locations with low WSS, high OSI, and high ECAP are prone to thrombus formation and have a higher risk of rupture [213,226]. On the other hand, in some studies reporting contradictory results [1,11,12,13], it is stated that low WSS and high OSI regions do not necessarily coincide with thrombus deposition and atherosclerosis formation [74,144,177].

Several studies in the literature [227,240] remarked on the limitations of WSS descriptors as scalar-tensor fields lacking directional information. These descriptors such as OSI, ECAP, and RRT can only indicate potential pathology but not possible progress, nor its underlying mechanisms [240]. Saqr et al. [227] highlights the utilization of vector fields, such as vorticity, to visualize hemodynamics. The utilization of vortex fields may afford a more comprehensive understanding of ILT development and rupture mechanisms, compared to WSS descriptors [227]. While the effect of wall behavior on WSS parameters has been extensively studied, there is limited research on wall deformation and vortex interactions within the AAA lumen. Kelsey et al. [213] stated that low-velocity recirculation zones are located near the regions where ILT has formed. Varble et al. [241] observed a significant correlation between intracranial aneurysm (IA) rupture and the near-wall vortical patterns exhibiting elevated vorticity levels. Biasetti et al. [74,177] correlated vortical structures with high WSS and attributed the bursting of the vortical structures to thrombus deposition at low WSS areas. Several studies have examined the relationship between the WSS parameters and vortex structures [228,242]. Zhan et al. [243] utilized the rortex as the vortex identification technique and found a positive correlation between vortices and high WSS regions. However, their study was limited by using zero pressure at the outlet and rigid wall boundary conditions.

## 7. Potential for Integrating AI and ML in AAA Research

Recent developments in artificial intelligence (AI) have influenced medicine. For that reason, the use of machine learning (ML) and data-driven approaches in AAA diagnosis and treatment is becoming increasingly common. By reducing manual interventions in modeling and analysis processes, ML methods can eliminate the risk of human error. Reducing the time required for analyses that may take weeks or days to order of hours or even real-time by ensuring faster execution of risk assessment processes is one of the advantages of data-driven methods.

ML describes the ability of systems to automatically learn from problem-specific data, providing a powerful alternative to manually building analytical models and enabling the automation of complex tasks. In recent years, the use of ML has rapidly expanded beyond computer science, including medical simulations [244], and ML has made great strides in sophisticated learning algorithms and efficient pre-processing techniques, such as the naissance of deep learning (DL), which represents the evolution of artificial neural networks (ANNs) towards deeper neural network architectures and the improvement of their learning capabilities [245]. DL methods are based on representation learning, transforming raw data into abstract and complex functions [245].

DL methods are used to improve computationally intensive methods, such as FEA and CFDs, making biomechanical simulations faster and more efficient. It has been demonstrated [246] that a trained DL model can predict stress distributions, with average errors of 0.492% in the von Mises stress distribution and 0.891% in peak von Mises stress, significantly reducing the long computing times typically required by FEA. This approach draws attention to DL as an alternative for FEA and CFDs in biomechanical and stress analyses, rapidly advancing patient-specific modeling in time-sensitive clinical applications. Similarly, in AAA rupture risk assessment, the generalized additive model has shown high accuracy in analyzing geometric and biomechanical markers for risk prediction [14].

The paradigm of ML and DL is the development of data-driven algorithms. Either structured or unstructured data are utilized to collect and derive the necessary task-related information. DL models have an advantage over traditional ML models due to their increased number of learning layers and higher abstraction levels on large data sets and complex insights [247]. Consequently, data-driven methods, particularly those utilizing deep learning architectures, are increasingly used in AAA diagnosis and treatment studies.

Deep architecture, composed of many nonlinear transformations, can compactly represent highly complex functions [248]. Deep neural networks (DNNs) are multi-layered artificial neural networks (ANNs) that can represent complex functions and learn complicated patterns more efficiently with fewer parameters, compared to shallow networks. Convolutional neural networks (CNNs) are analogous to traditional ANNs, as they consist of neurons that optimize themselves through learning, but they are particularly effective in pattern recognition tasks involving images [249]. CNNs reduce the need for fully connected layers by focusing on learning in convolutional layers, which decreases computational cost and the number of parameters [250].

CNNs have gained significant popularity in computer vision due to their ability to efficiently process spatial information, compared to fully connected architectures, and have become a preferred method for medical imaging tasks [251,252]. Deep convolutional neural networks (DCNNs) concentrate on learning in convolutional layers, reducing the need for fully connected layers, which lowers the number of parameters and computational costs [250]. These networks use local connections to identify image features like edges, corners, and textures, enabling better interpretation of spatial information [253].

**Figure 11:**
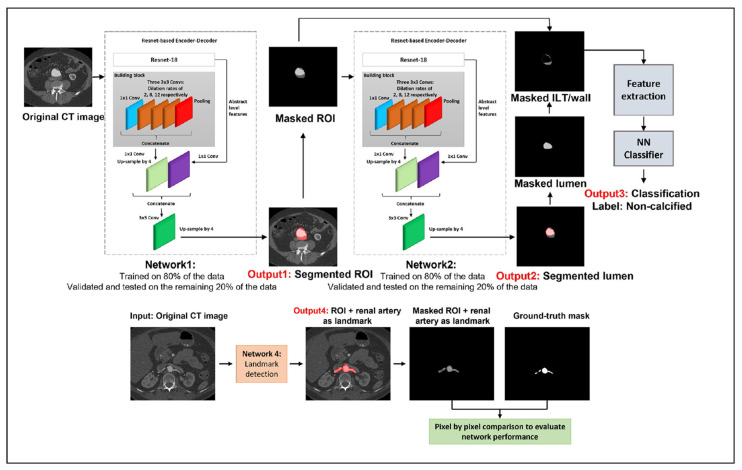
Flowchart of the Resnet FCN architecture and AAA segmentation outputs. Adapted from [63] under the terms of the CC BY 4.0 license.

Segmentation processes for AAA simulations, whether manual or semi-automatic, have benefited from advancements in image processing algorithms, large datasets, and DL techniques. DL-based segmentation methods, such as ARU-Net [49] and CACU-Net [50], have reduced the time to generate computational models from computed tomography angiography (CTA) scans from approximately 2 h to 10 min, showing reliable performance in creating patient-specific geometries for CFDs simulations [254]. Figure 11 illustrates the fully automatic segmentation of AAAs from CT images by adapting the Resnet-based fully convolutional networks (FCNs) [63]. The model consists of three steps, extracting the aorta and iliac arteries, and detecting the lumen and other AAA tissues. The automated segmentation results demonstrate a good agreement with manual segmentation. DCNNs achieved a dice similarity of 82% in segmenting intraluminal thrombus (ILT), showing that AI-based segmentation has the potential to be used for clinical applications, especially in cases with difficult-to-detect boundaries [255].

### Applications of AI and ML in AAA Flow Simulations

The application of AI and ML in AAA simulations has gained considerable attention, as several studies have demonstrated their potential in improving both speed and accuracy in predicting hemodynamic parameters and disease progression. For instance, Liang et al. [256,257] showed that DNNs, trained on hemodynamic data derived from CFDs simulations, could predict steady-state distributions of pressure, velocity, and velocity magnitude within one second. This achievement highlights the capacity of DNNs to act as rapid surrogates for traditional CFDs simulations, significantly reducing computational time. Rościszewski et al. [258] also explored the integration of AI to accelerate CFDs simulations, emphasizing the importance of such techniques in clinical settings where fast and accurate predictions are critical. As demonstrated in studies such as Feiger et al. [259] and Hahn et al. [260], at least nine simulations were necessary to train neural networks to predict time-averaged WSS and pressure gradients across stenotic regions, illustrating how ML can be combined with CFDs and FEA simulations to develop precise and patient-specific flow prediction models [51]. Similarly, Kim et al. [261] applied neural network architectures to a dataset of 54 patients, testing the model on four key features: radius, ILT thickness, time-averaged WSS, and aneurysm growth rate.

Recurrent neural network (RNN)- and CNN-based approaches have also been widely used for the prediction of future events in medical applications [262,263]. CFDs simulations conducted on patient-specific 3D geometries show that DL frameworks, trained with multi-physical features to overcome small amounts of longitudinal AAA data, can outperform traditional methods in predicting AAA growth with improving accuracy and efficiency. These improvements can help clinicians manage AAA progression and improve patient care [261]. Jiang et al. [264] employed a combination of physical vascular adaptation modeling, machine learning (ML) tools, and follow-up scan data to predict the shape evolution of AAAs using a Deep Belief Network (DBN).

Soudah et al. [265] demonstrated a multilayer perceptron (MLP) based approach, creating two neural networks for AAA analysis. The first, a Mesh Neural Network (MNN), generates the aneurysm geometry based on four geometric factors, while the second, the Tension Neural Network, calculates maximum wall stress by combining the MNN output with the arterial pressure, achieving an accuracy rate of 95% compared to the finite element method (FEM)-based results. However, as MNN results can be noisy due to uneven node distribution, further studies are necessary to account for factors such as ILT effects. Additionally, statistical shape models (SSMs) have been used to describe the shape variability of the aortic arch, with nonlinear regression employed to analyze pressure gradients as a function of flow rate and cross-sectional area [199,260,266].

Joly et al. [199] utilized longitudinal cohort data from CFDs simulations and CT scans to study hemodynamic correlations, highlighting ML’s potential to handle complex biomechanical phenomena. However, the unpredictable nature of fluid dynamics can negatively affect the AI predictions, which is why combining AI-driven mechanisms with FEM could improve the accuracy of models. For example, Jiang et al. [267] adopted this hybrid approach to estimate aneurysm growth in the aorta, achieving greater accuracy while reducing computation time through optimization techniques and AI.

Recent advancements in AI have also enhanced MRI-based blood flow measurements, particularly with 4D flow MRI, as ML models like CNNs and U-Nets have been applied to automate tasks, such as phase-contrast imaging, vessel segmentation, and contour drawing [268]. ML-based super-resolution techniques have been developed to integrate high-resolution CFDs simulations with MRI data, resulting in more realistic flow simulations without sacrificing data fidelity [269].

In addition to these improvements and advantages, there are various challenges to overcome, such as choosing the right implementation, bias and drifting in data, and the mitigation of black-box properties [270]. Further research is necessary to refine AI models for broader clinical applications. For example, CNNs used for segmentation still require significant improvements before they can be widely adopted in clinical settings [254]. Additionally, future research could focus on retraining DNNs using datasets generated by more advanced simulation techniques, such as FSI, to enhance model accuracy and clinical relevance. Exploring larger datasets and more complex architectures could further enhance the predictive capabilities of AI models. Given the scalability and rapid computation time of DNNs, they hold great promise for future clinical applications [256].

Despite the significant advances in machine learning (ML) for AAA analysis, several limitations remain in terms of clinical translation and reliability. A major challenge is the heterogeneity in medical imaging quality and the limited availability of large, high-quality datasets. Many ML models are trained on retrospective data with imbalanced sample distributions, which can lead to biases and reduced generalizability across diverse patient populations [51,271]. This issue is particularly evident in automatic segmentation algorithms, which have been shown to overestimate intraluminal thrombus (ILT) volumes, impacting clinical decision-making [51].

Another key limitation is the “black box” nature of deep learning models, making their predictions difficult to interpret in clinical settings [272,273]. The lack of transparency in ML decision-making processes poses a barrier to widespread adoption, as clinicians require explainable outputs to support patient care. Furthermore, the high computational demands of ML algorithms, particularly those integrating complex biomechanical simulations, can limit their practical use in real-time applications [274,275].

The risk of overfitting is another concern, as many ML models are developed using small, domain-specific datasets that may not adequately represent broader patient populations [275]. This challenge is further compounded by medical “hallucinations”—false or misleading outputs generated by AI models—emphasizing the critical need for rigorous validation using large, diverse, and prospectively collected datasets [275].

Additionally, while ML has demonstrated potential in AAA risk prediction and CFDs integration, the fusion of data-driven methods with traditional FEA and FSI simulations remains an ongoing research area. Hybrid approaches necessitate further validation to confirm their reliability and clinical relevance. Moreover, regulatory barriers, particularly the requirement for comprehensive validation and approval processes, remain significant obstacles to the integration of ML-based AAA assessment tools into clinical practice.

## 8. Conclusions

In this paper, we focused on modeling realistic BCs and the application of ML techniques for AAAs, providing a comprehensive overview and summarizing their implications. The main conclusions of the study are provided below.

The numerical models developed to simulate a specific region of the cardiovascular system could accurately reproduce patient-specific hemodynamics and biomechanics in a virtual environment, keeping the computational time within reasonable limits. To accurately introduce the hemodynamic properties at the upstream and downstream areas of the specific region of interest, various BCs have been developed. These conditions, known as inlet and outlet BCs, are artificial boundaries that don’t correspond to real boundaries in the original system. They play a crucial role in creating a realistic model of the entire cardiovascular system by accurately connecting the region of interest to the rest of the system while reducing computational demand. On the other hand, the artery wall is a physical boundary in the cardiovascular models. To accurately describe wall deformation under hemodynamic loads, the FSI technique is generally implemented at the solid–fluid interface. This method couples CFDs simulation of hemodynamics with FEA simulation of wall mechanics. To precisely characterize the mechanical properties of the artery wall, researchers have developed constitutive models based on tensile tests of the arterial tissue.

Across multiple studies, certain patterns have emerged. Hemodynamic and biomechanical parameters, such as TAWSS, OSI, ECAP, RRT, PWS, and wall displacement, are highly sensitive to the selected boundary conditions at the inlet, outlet, and wall. In the FSI studies of AAAs with deformable walls, generally, a time-dependent flow rate at the IR section of the aorta is supplied as a fully developed, parabolic velocity profile at the inlet. The prescribed time-dependent pressure profile is given downstream of the external and internal iliac arteries. More sophisticated BCs at the inlet and outlet, such as the patient-specific, Womersley profile, or 3-element Windkessel models are generally implemented in pure hemodynamics studies, neglecting wall deformation by implementing rigid wall assumption, to decrease the model complexity and computational cost.

**Figure 12:**
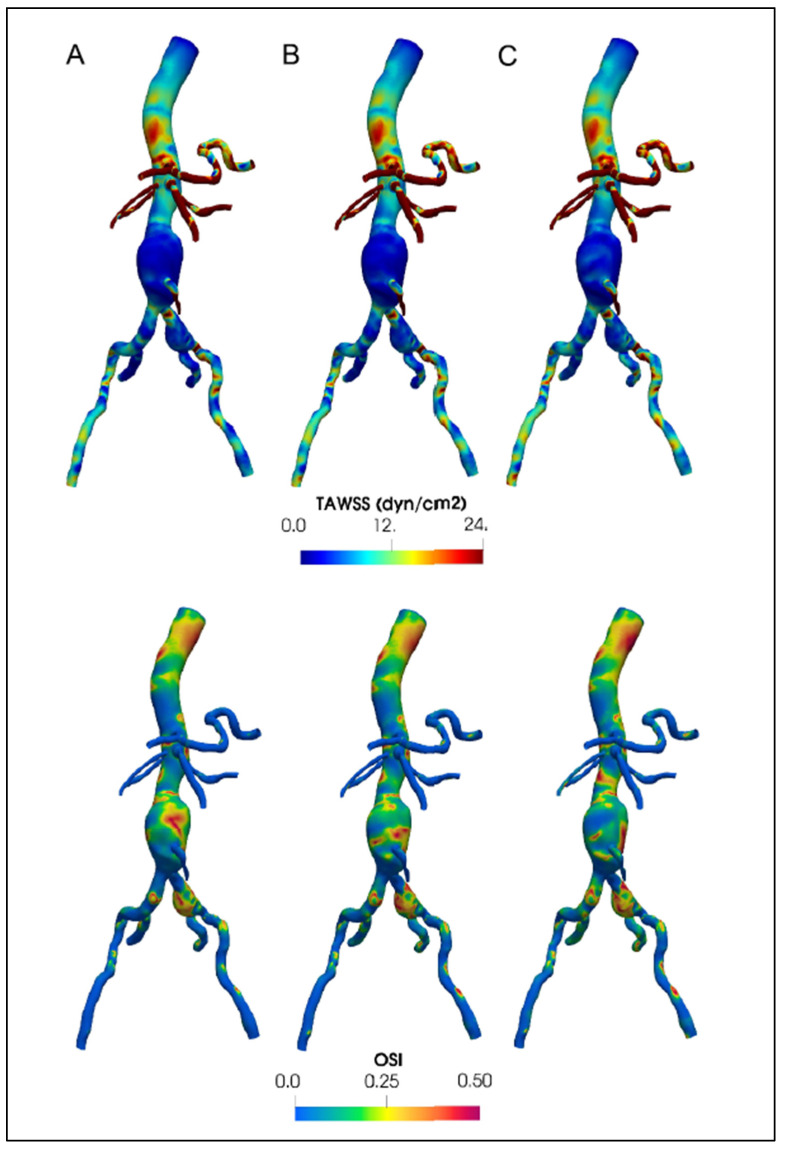
The time-averaged wall shear stress (TAWSS, top) and oscillatory shear index (OSI, bottom) for (A) E-uniaxial wall model, (B) E-biaxial wall model, and (C) rigid walls. Reproduced from [52] under the terms of the CC BY 4.0 license.

Despite the advancements in the integration of accurate BCs at the inlet, outlet, and wall, the integration and coupling of sophisticated BCs with various multiphysics and multidomain aspects is quite challenging. Defining patient-specific velocity profiles at the inlet boundary is limited due to the cardiac motion and resolution of measuring devices. Coupling the lumped parameter models to the outlets requires patient-specific parameter estimation that necessitates the development of different algorithms or prior CFDs simulations. The accurate integration of wall motion is possible with the proper discretization and solution of the governing equations of the mechanical domain using hyperelastic and anisotropic constitutive equations with patient-specific parameters, which requires excessive computational time. As an example, Lan et al. [52] have simulated the complete abdominal region from the SC zone by coupling the WK3 BC at the outlet and the FSI at the walls. In Figure 12, TAWSS and OSI comparisons of uniaxial, biaxial, and rigid wall models are illustrated. The simulation was performed over three cardiac cycles, that take nearly 15 h using three Intel Xeon Gold 5118 processors interconnected by a 100 GB/s EDR InfiniBand for a total of 72 threads, operating at 191 GB RAM and a clock rate of 2.3 GHz. Qin et al. [186] modeled the complete aorta by dividing it into four partitions by implementing the Newton–Krylov–Schwartz algorithm, assuming the walls as rigid and with a resistance BC at the outlet. They reported computational time as 87 h for 2880 cores for $30.06\times 10^{6}$ elements with nearly 70% parallel efficiency, even without extensive coupling of the FSI and WK3. Later, they applied a highly parallel framework to study the effect of primary and peripheral branches on the local hemodynamics of the abdominal aorta [185]. The results demonstrate that the peripheral branches significantly affect the flow field within the AAA, which emphasizes the significance of the complete modeling of the AAA domain, by focusing on the portion that starts from the SC and accounts for at least the primary branches, such as CT, SM, LR, RR, ARR, LCI, and RCI arteries. However, the AAA studies with deformable wall BCs typically start from the IR region, by eliminating upper arterial branches to prevent the excessive computational cost. Therefore, most of them utilize prescribed pressure waveform BCs at the outlets, which are located downstream of the left and right iliac arteries. Lan et al. [52] utilized a patient-specific computational domain with elastic and isotropic deformable walls, of which the inlet section started from the SC region, and the WK3 outlet boundary condition was implemented. Table 3 presents a structural comparative analysis of the accuracy, computational cost, and clinical relevance of each method.

To the best of the author’s knowledge, the current literature lacks a model that incorporates the FSI at the walls, begins from the supraceliac (SC) region, using 4D PC-MRI measured patient-specific inlet velocity profiles, and implements the WK3 outlet BCs at the aorta branches. Furthermore, the vast majority of the studies using the FSI together with the WK3 implement hyperelastic and isotropic constitutive models, rather than models accounting for wall anisotropy. Despite the significant advancements in computational models for AAA biomechanics, tuning the wall compliance parameter, C, of the WK3 model compatible with the hyperelastic and anisotropic nature of arterial walls is missing in the existing literature. Therefore, developing a method for the tuning of the WK3 parameters to reflect the wall hyperelasticity and anisotropy would be highly valuable for better understanding and predicting the behavior of arterial flow, wave propagation, and wall mechanics in this region.

The primary purpose of the research focusing on AAA hemodynamics and biomechanics is to provide clinicians with a tool that assists them in understanding the growth and potential rupture of the aneurysm during the surgical decision-making process. However, it is still a big challenge to accurately resolve the flow field and wall mechanics within a clinically acceptable computing time. Moreover, the limited resolution of medical imaging and measuring devices prevents accurate description of the local time-dependent velocity profiles and pressure waveforms, patient-specific geometry, and wall thickness. These factors limit their practical use in real-time clinical practice, where timely interventions are crucial. Therefore, comparative studies are required to evaluate how simplified and less time-consuming models perform relative to their more accurate, computationally intensive counterparts. To integrate computational models into clinical workflow, the development of simplified models that are validated against more accurate and patient-specific models is necessary. At that point, the ML tools and data-driven techniques might be quite helpful in providing clinical predictions due to their efficiency. However, to develop a reliable ML model, highly accurate CFDs data sets should be produced by improving the precision of the modeling parameters.

The reliability of computational models in AAA hemodynamics and biomechanics depends on their capacity to consider patient variability and clinical validation. While computational models have significantly improved with patient-specific geometries and BCs, validation against in vivo data is crucial. Studies using 4D flow MRI patient-specific inflow velocity profiles predict different WSS and PWS compared to artificial profiles, which highlights the importance of integrating clinical data. Although validating CFDs results with in vivo measurements remains challenging due to limitations in pressure and stress measurements using PC-MRI, comparative studies reported sufficient similarity between flow fields obtained using in vivo measurements and CFDs models with sophisticated BCs tuned with patient-specific data.

Variations in cardiac output, blood pressure, viscosity, and vascular compliance among AAA patients have substantial impacts on simulation results. Although there are studies that implemented patient-specific inflow conditions, WK3 parameters tuning, rheology, and wall material properties, many studies relied on simplified boundary conditions due to their efficiency and accessibility, which may not resolve patient-specific variations and create problems in clinical applications.

Additionally, ML models seem to be potential surrogates for CFDs/FEA models in clinical applications, primarily trained and tested by simulation-based datasets. This is due to their ability to efficiently process large volumes of data and identify complex patterns that might be overlooked by traditional methods. Training and testing data generated with simplified BCs may reduce the precision of their predictions by introducing additional biases and blocking physiological diversity. Therefore, the comprehensive assessment of both validation and patient variability is essential in integrating these advanced ML techniques into clinical workflows, and this can result in more personalized and accurate treatment plans, ultimately improving patient outcomes and healthcare efficiency.

## Figures and Tables

**Table 1:** Boundaries of fluid and solid domains of AAA models.

Boundary Type (Γ)	Domain (Ω)	
	$\mathrm{Fluid}\;{(\Omega }_{f})$	$\mathrm{Solid}\;(\Omega_{s})$
Inlet	$\Gamma_{in}^{F}$	$\Gamma_{in}^{S}$
Outlet $\left(i^{th}\right)$	$\Gamma_{out,i}^{F}$	$\Gamma_{out,i}^{S}$
FSI Interface	$\Gamma_{FSI}^{F}$	$\Gamma_{FSI}^{S}$
External Wall	−	$\Gamma_{ext-wall}^{S}$

**Table 2:** Flow-split rates through branch arteries.

Artery Branch	Flow Split
$Q^{CT}$	$0.33(Q^{SC}-Q^{IR})$
$Q^{LR},\;Q^{RR}{,Q}^{SMA}$	$0.223(Q^{SC}-Q^{IR})$
$Q^{LEI},Q^{REI}$	$0.7Q^{IR}$
$Q^{LII},Q^{RII}$	$0.3Q^{IR}$
$Q^{S},Q^{H}$	${0.5Q}^{CT}$

**Table 3:** A structural comparative analysis between accuracy, computational cost, and clinical relevance of each method.

BC Model	Accuracy	Computational Cost	Clinical Relevance
Uniform and Parabolic Inlet Profiles	(L) Does not capture patient-specific flow variations and pulsatile nature of blood flow	(L) Not costly	(L) Simplified models may not reflect realistic hemodynamics
Womersley Inlet Profile	(H) Captures pulsatile nature of blood flow	(M) Requires analytical computation of velocity distribution	(M) Common in cardiovascular studies but may not reflect patient-specific hemodynamics
4D Flow MRI-based Inlet	(VH) Patient-specific and time-dependent	(VH) Computationally expensive and data-intensive	(H) Clinically relevant, but limited availability due to imaging constraints
Prescribed Pressure Outlet	(M) Assumes static or average pressure conditions	(L) Simple to implement and computationally efficient	(M) Common in AAA studies but does not reflect accurate wave propagation characteristics of vessels
3-Element Windkessel (WK3) Outlet	(H) Models vascular resistance, compliance and pressure reflections	(H) Requires parameter tuning and iterative solutions	(H) Clinically relevant when patient-specific parameters are available
FSI with Elastic Wall	(H) Captures wall deformation and ILT effects	(VH) Requires coupling between CFDs and FEA, increasing computational time	(H) Improves stress predictions but difficult to integrate clinically
FSI with Hyperelastic and Anisotropic Wall	(VH) Most realistic representation of arterial wall and ILT mechanics	(EH) Very expensive especially for large-scale studies	(H) Needed for advanced biomechanical analysis, but not practical for routine clinical use

(L) Low (M) Medium (H) High (VH) Very High (EH) Extremely High.

## Data Availability

Not applicable.

## Supplementary Materials

The following supporting information on derivation, algorithm and code of Womersley velocity profile inlet boundary condition can be downloaded at: https://www.mdpi.com/article/10.3390/bioengineering12050437/s1.

## Abbreviations

The following abbreviations are used in this manuscript:

AAA	Abdominal aortic aneurysm
ILT	Intraluminal thrombus
CFDs	Computational fluid dynamics
FEA	Finite element analysis
FSI	Fluid–structure interaction
BC	Boundary condition
WK2	2-element Windkessel model
WK3	3-element Windkessel model
WK4	4-element Windkessel model
RC	2-element Windkessel model
RCR	3-element Windkessel model
RLC	4-element Windkessel model
RCR	3-element Windkessel model
WSS	Wall shear stress
TAWSS	Time-averaged wall shear stress
OSI	Oscillatory shear index
ECAP	Endothelial cell activation potential
RRT	Relative residence time
IR	Infrarenal region
SC	Supraceliac region
CT	Celiac trunk branch
H	Hepatic
SM	Superior mesenteric
LR	Left renal
RR	Right renal
ARR	Accessory renal
LEI	Left external iliac
REI	Right external iliac
LII	Left internal iliac
RII	Right internal iliac
ML	Machine learning
DL	Deep learning

## Appendix A

Different choices for the function $F\left(\dot{\gamma }\right)$ correspond to different constitutive models for blood, with material constants depending on factors such as temperature, hematocrit, and plasma. In the literature, eight non-Newtonian models are commonly used to represent the shear-thinning behavior of blood: Carreau, Carreau–Yasuda [72,73,74,75,76], Casson, Quemada [77], Power, Cross, Simplified Cross, and Modified Cross [76,78,79,80,81,82,83,84,85,86], as summarized in Table A1.

**Table A1:** Selected shear-thinning rheology models, which are frequently used to model blood [71,84,86,276,277].

**Model Name**	**Equation**	**Constants**
Carreau	$\frac{\mu \left(\dot{\gamma }\right)-\mu_{\infty }}{\mu_{0}-\mu_{\infty }}={\left(1+{\left(\lambda \dot{\gamma }\right)}^{2}\right)}^{(n-1)/2}$	$\mu_{0}=0.056\;Pa\cdot s$
		$\mu_{\infty }=0.00345\;Pa\cdot s$
		λ=3.313 s
		n =0.3568
Carreau–Yasuda	$\frac{\mu \left(\dot{\gamma }\right)-\mu_{\infty }}{\mu_{0}-\mu_{\infty }}={\left(1+{\left(\lambda \dot{\gamma }\right)}^{a}\right)}^{(n-1)/a}$	$\mu_{0}=0.056\;Pa\cdot s$
		$\mu_{\infty }=0.00345\;Pa\cdot s$
		λ =1.902 s
		n =0.22
		a=1.25
Quemada	$\mu =\mu_{f}\left[1-\frac{1}{2}\frac{K_{0}+K_{\infty }\sqrt{\frac{\left|\dot{\gamma }\right|}{\gamma_{c}}}}{1+\sqrt{\frac{\left|\dot{\gamma }\right|}{\gamma_{c}}}}\phi \right]$	$\mu_{f}=0.0012\;\;Pa\cdot s$
		$K_{0}=4.65$
		$K_{\infty }=1.84$
		$\gamma_{c}=2.23\;s^{-1}$
		ϕ=0.4
Casson	$\sqrt{\tau }=\sqrt{k_{0}}+\sqrt{k_{1}\dot{\gamma }}$	$k_{0}=0.05\;dyne/{cm}^{2}$
		$k_{1}=0.04\;dyne/{cm}^{2}$
Cross	$\frac{\mu \left(\dot{\gamma }\right)-\mu_{\infty }}{\mu_{0}-\mu_{\infty }}=\frac{1}{1+{\left(m\dot{\gamma }\right)}^{n}}$	$\mu_{0}=0.056\;Pa\cdot s$
		$\mu_{\infty }=0.00345\;Pa\cdot s$
		m=1.007 s
		n=1.028
Simplified Cross	$\frac{\mu \left(\dot{\gamma }\right)-\mu_{\infty }}{\mu_{0}-\mu_{\infty }}=\frac{1}{1+m\dot{\gamma }}$	$\mu_{0}=0.103\;Pa\cdot s$
		$\mu_{\infty }=0.005\;Pa\cdot s$
		m=8 s
Modified Cross	$\frac{\mu \left(\dot{\gamma }\right)-\mu_{\infty }}{\mu_{0}-\mu_{\infty }}=\frac{1}{{\left(1+{\left(m\dot{\gamma }\right)}^{n}\right)}^{a}}$	$\mu_{0}=0.056\;Pa\cdot s$
		$\mu_{\infty }=0.00345\;Pa\cdot s$
		m=3.736 s
		n=2.406
		a=0.254
Power	$\mu \left(\dot{\gamma }\right)=K{\dot{\gamma }}^{n-1}$	$K=0.017\;Pa\cdot s^{n}$
		n=0.708 [277]
		and
		$K=0.035\;Pa\cdot s^{n}$
		n=0.6 [84]

## Appendix B

The calculation of the local from the global WK3 parameters for the downstream CT, SMA, LR, RR, LCI, and RCI compartments is summarized in Table A2.

**Table A2:** Calculation of the local from the global WK3 parameters for the downstream CT, SMA, LR, RR, LCI, and RCI compartments, using 1D equations and Murray’s Law.

**Global WK3**	$R_{T}^{SC}$	$(P_{mean}^{SC}-P_{out})/{\bar{Q}}^{SC}$
	$C^{SC}$	${(Q}_{max}^{SC}-Q_{min}^{SC})\cdot \Delta t/{(P}_{sys}^{SC}-P_{dia}^{SC})$
$\Gamma_{out}^{CT}$	$C^{CT}$	${(\bar{Q}}^{CT}/{\bar{Q}}^{SC})\cdot C^{SC}$
	$R_{T}^{CT}$	${(\bar{Q}}^{SC}/{\bar{Q}}^{CT})\cdot R_{T}^{SC}$
	$R_{p}^{CT}$	$0.056\cdot R_{T}^{CT}$
	$R_{d}^{CT}$	$0.994\cdot R_{T}^{CT}$
$\Gamma_{out}^{SMA}$	$C^{SMA}$	${(\bar{Q}}^{SMA}/{\bar{Q}}^{SC})\cdot C^{SC}$
	$R_{T}^{SMA}$	${(\bar{Q}}^{SC}/{\bar{Q}}^{SMA})\cdot R_{T}^{SC}$
	$R_{p}^{SMA}$	$0.056\cdot R_{T}^{SMA}$
	$R_{d}^{SMA}$	$0.994\cdot R_{T}^{SMA}$
$\Gamma_{out}^{LR,RR}$	$C^{LR,RR}$	${(\bar{Q}}^{LR,RR}/{\bar{Q}}^{SC})\cdot C^{SC}$
	$R_{T}^{LR,RR}$	${(\bar{Q}}^{SC}/{\bar{Q}}^{LR,RR})\cdot R_{T}^{SC}$
	$R_{p}^{LR,RR}$	$0.28\cdot R_{T}^{LR,RR}$
	$R_{d}^{LR,RR}$	$0.72\cdot R_{T}^{LR,RR}$
$\Gamma_{out}^{LCI,RCI}$	$C^{LCI,RCI}$	${(\bar{Q}}^{LCI,RCI}/{\bar{Q}}^{SC})\cdot C^{SC}$
	$R_{T}^{LCI,RCI}$	${(\bar{Q}}^{SC}/{\bar{Q}}^{LCI,RCI})\cdot R_{T}^{SC}$
	$R_{p}^{LCI,RCI}$	$0.056\cdot R_{T}^{LCI,RCI}$
	$R_{d}^{LCI,RCI}$	$0.994\cdot R_{T}^{LCI,RCI}$

## Appendix C

In the literature, different vortex identification methods have been proposed. The most well-known vortex identification methods are Q-criterion, Δ-criterion, $\lambda_{2}$-criterion, and $\lambda_{ci}$-criterion. All of these methods require different requirements to define a vortex. *Q*-criterion, Δ-criterion, and $\lambda_{ci}$-criterion are called velocity gradient-based vortex identification criteria, while $\lambda_{2}$-criterion utilizes the local pressure minimum to define a vortex.

Velocity gradient tensor D=∇v can be partitioned into symmetric and antisymmetric parts as follows:

(A1) D=ε+Ω 

(A2) $$\varepsilon =1/2\left[D+D^{T}\right]\;\;$$

(A3) $$\Omega =1/2\left[D-D^{T}\right]\;$$

where ε and Ω are the strain rate and angular rotation rate tensors, respectively. The trajectory of a fluid particle can be determined by solving the following differential equation:

(A4) $$\frac{d\vec{y}}{dt}=D\cdot \vec{y}$$

with an initial condition $y\left(0\right)=y_{0}.$ The eigendecomposition of the above ordinary differential equation can be written as

(A5) $$D=V\Lambda V^{-1}$$

where the columns of V are eigenvectors $v_{i},$ and the diagonal of Λ gives the eigenvalues $\lambda_{i}$. The general solution of the above equation is

(A6) $$y\left(t\right)=\nu_{1}\exp\left(\lambda_{1}t\right)c_{1}+\nu_{2}\exp\left(\lambda_{2}t\right)c_{2}+\nu_{3}\exp\left(\lambda_{3}t\right)c_{3}$$

where constants $c_{i}$ are determined from the initial condition. According to the above equation, if all eigenvalues are real, then the point obtained is called a node or a saddle. However, if one of the eigenvalues is real but the other two are complex conjugates, the point obtained becomes a focus, with closed or spiraling streamline trajectories, which is an indication of a vortex.

To find the eigenvalues of the diagonal, characteristic equations can be solved as follows:

(A7) $$det\left[\frac{\partial u_{i}}{\partial x_{j}}-\lambda I\right]=0$$

which obtains

(A8) $$\lambda^{3}+P\lambda^{2}+Q\lambda +R=0$$

(A9) $$P=-tr\left(D\right)$$

(A10) $$Q=\frac{1}{2}\left({\left[tr(D)\right]}^{2}-tr(D^{2})\right)=\frac{1}{2}\left({\left[tr(D)\right]}^{2}+{\left\|\Omega \right\|}^{2}-{\left\|\varepsilon \right\|}^{2}\right)$$

(A11) $$R=-det\left(D\right)=\frac{1}{3}\left(-P^{3}+3PQ-tr\left(D^{3}\right)\right)$$

where P, Q, and R are invariants of the velocity gradient tensor [229]. For an incompressible flow, $tr\left(D\right)$ is zero. The discriminant of the characteristic equation gives an idea of whether the eigenvalues are real or complex, which can be written as

(A12) $$\Delta ={\left(Q/3\right)}^{3}+{\left(R/2\right)}^{2}$$

If Δ≥0, one eigenvalue is real, and the other two eigenvalues are complex conjugates, which indicates the swirling flow having a spiraling or closed streamline pattern, and this is called as Δ-criterion. Q is the second invariant of the velocity gradient tensor, and for an incompressible flow, it is written as

(A13) $$Q=\frac{1}{2}\left({\left\|\Omega \right\|}^{2}-{\left\|\varepsilon \right\|}^{2}\right)>0$$

Zhou et al. [278] also utilized the complex eigenvalue of the velocity gradient tensor to visualize vortices. For that purpose, they decompose the velocity gradient tensor with the help of diagonalization by using eigenvalues and eigenvectors, as follows:

(A14) $$\frac{{\partial u}_{i}}{{\partial x}_{j}}=\left[v_{r}\;\;\;v_{cr}\;\;\;v_{ci}\right]\left[\begin{array}{ccc} \lambda_{r} & 0 & 0\\ 0 & \lambda_{cr} & \lambda_{ci}\\ 0 & {-\lambda }_{ci} & \lambda_{cr} \end{array}\right]{\left[v_{r}\;\;\;v_{cr}\;\;\;v_{ci}\right]}^{-1}$$

where $\lambda_{r}$ is the real eigenvalue with as corresponding real eigenvector $v_{r}$, and $\lambda_{cr}\pm \lambda_{ci}i$ are the conjugate pair of the complex eigenvalues with complex eigenvectors $v_{cr}\pm \;v_{ci}i$. The local streamlines can be expressed in a local coordinate as

(A15) $$y_{1}\left(t\right)=C_{r}\exp\left(\lambda_{r}t\right)$$

(A16) $$y_{2}\left(t\right)=exp\left(\lambda_{cr}t\left[{C_{c}}^{1}\cos\left(\lambda_{ci}t\right)+{C_{c}}^{2}\sin\left(\lambda_{ci}t\right)\right]\right)$$

(A17) $$y_{3}\left(t\right)=\exp\left(\lambda_{cr}t\left[{C_{c}}^{2}\cos\left(\lambda_{ci}t\right)-{C_{c}}^{1}\sin\left(\lambda_{ci}t\right)\right]\right)$$

where $C_{r},$ ${C_{c}}^{1}$, and ${C_{c}}^{2}$ are constants and can be determined from initial conditions. Along the axis of eigenvector $v_{r}$, the flow is either stretched or compressed, while the flow is swirling on the plane of eigenvectors $v_{cr}$ and $v_{ci}$. Hence, the imaginary parts, $\lambda_{ci},$ of the complex eigenvalues might be evaluated as a vortex indicator, and connected regions having non-zero $\lambda_{ci}$ can be specified as vortex structures. The complex eigenvector, $\lambda_{ci}$, is known as the strength of the swirling motion and the swirling strength of a vortex.

The $\lambda_{2}$-criterion is based on local pressure minimum across a vortex core. Jeong and Hussain [279] derived a transport equation for the strain rate tensor by taking the gradient of the incompressible Navier–Stokes equation, and obtaining the symmetric part, as follows:

(A18) $$\frac{d\varepsilon }{dt}+\nu \nabla^{2}\varepsilon +\varepsilon^{2}+\Omega^{2}=-\frac{1}{\rho }\nabla \left(\nabla P\right)$$

The term at the right-hand side is called the pressure Hessian matrix. According to the $\lambda_{2}$-criterion, the vortex core is located at the local pressure minimum, except in the presence of unsteady and viscous effects. Therefore, by neglecting the rate of change in the irrotational straining and viscous effects, the pressure Hessian can be found as

(A19) $$\varepsilon^{2}+\Omega^{2}\approx -\frac{1}{\rho }\nabla \left(\nabla P\right)$$

The local pressure minimum can be obtained where the pressure Hessian has two positive eigenvalues, which requires the $\varepsilon^{2}+\Omega^{2}$ term to have two negative eigenvalues. In 3D flows, ordering the eigenvalues of this matrix, to satisfy both, must be negative inside a vortex core, as follows:

(A20) $$\lambda_{1}\leq \lambda_{2}\leq \lambda_{3\;\;\;}\&\;\;\;\;\;\lambda_{2}<0\;\;$$

In 2D flows, $\lambda_{1}\leq \lambda_{2}$ should be two eigenvalues of the matrix. According to the $\lambda_{2}$-criterion, $\lambda_{2}$ must be negative, which identifies the connected vortex zones.
