# Supplementary material for: Modeling Techniques and Boundary Conditions in Abdominal Aortic Aneurysm Analysis: Latest Developments in Simulation and Integration of Machine Learning and Data-Driven Approaches
Source: Bioengineering (Basel). 2025 Apr 22;12(5):437. doi: 10.3390/bioengineering12050437 (PMC12108684; doi:10.3390/bioengineering12050437)
Supplement: Supplementary file 1 [file bioengineering-12-00437-s001.zip › bioengineering-3536850-supplementary.pdf]

Supplementary Material

Womersley Velocity Profile Boundary Condition

Over several decades, intense research on physiological flow inside the arteries has been performed. In 1955, Womersley [1] derived the exact solution of incompressible, Newtonian fluid flow through a cylindrical and rigid blood vessel, in which a pressure gradient which is periodic function of time drives the flow, by using the equations of motion and continuity. Assuming the motion is laminar, axisymmetric, and parallel to the longitudinal axis of the tube in addition to the vessel is horizontal, and gravitation has no effect on the flow, the Navier-Stokes equations, and the equation of continuity are simplified to the following under the conditions named above:

$$\frac{\partial u}{\partial x} = 0 \qquad (S1)$$

$$0 = -\frac{\partial p}{\partial r} \qquad (S2)$$

$$0 = -\frac{\partial p}{\partial \theta} \qquad (S3)$$

$$\rho\frac{\partial u}{\partial t} = -\frac{\partial p}{\partial x} + \mu\left(\frac{\partial^2 u}{\partial r^2} + \frac{1}{r}\frac{\partial u}{\partial r}\right) \qquad (S4)$$

The boundary conditions are the axisymmetric condition at the center and no-slip on the wall, at radius R:

$$\frac{\partial u}{\partial r} = 0 \qquad at \quad r = 0 \qquad (S5)$$

$$u = 0 \qquad at \quad r = R \qquad (S6)$$

Here p is for pressure, x, r and θ are cylindrical polar coordinates with x in the axial and r in the radial direction, u is the velocity component in the direction of x, and t is time. According to Equations (S2) and (S3), p is a function of x and t only. According to Equation (S4), u is a function of r and t. On differentiating Equation (S4) with respect to x, one obtains

$$\frac{\partial}{\partial x}\left(\frac{\partial p}{\partial x}\right) = 0 \qquad (S7)$$

This shows that the pressure gradient must not vary with x. It can be a function of *t*. For simplicity, the form of the pressure gradient will be taken as a simple harmonic motion and written in complex form:

$$\frac{\partial \mathrm{p}}{\partial \mathrm{x}} = \mathrm{Ae}^{\mathrm{in}\omega\mathrm{t}} \qquad (\mathrm{S8})$$

On substituting into Equation (S4), one obtains

$$\rho\frac{\partial u}{\partial t} = -Ae^{in\omega t} + \mu\left(\frac{\partial^2 u}{\partial r^2} + \frac{1}{r}\frac{\partial u}{\partial r}\right) \qquad (S9)$$

After solving Equation (S9) in accordance with the stated boundary conditions, one can obtain

$$u(r,t) = \frac{A}{i\omega\rho}\left[1 - \frac{J_0\left(\alpha\frac{r}{R}i^{3/2}\right)}{J_0(\alpha i^{3/2})}\right]e^{i\omega t} \qquad (S10)$$

where ν is kinematic viscosity and α is a dimensionless quantity known as the Womersley number

$$\alpha = R\sqrt{\frac{\omega}{\nu}} \qquad (S11)$$

If the flow rate inside the blood vessel is known, the term *A* can also be determined from the given formula

$$Q = 2\pi\int_0^R urdr \qquad (S12)$$

Substituting the u in Equation (S10) to flow rate integral and writing $y = r/R$,

$$Q = \frac{2\pi A}{i\omega\rho}\left(\frac{R^2}{2} - \frac{R^2}{J_0(\alpha i^{3/2})}\int_0^1 J_0\left(\alpha y i^{3/2}\right)ydy\right)e^{i\omega t} \qquad (S13)$$

From known properties of Bessel functions, $\int x\mathrm{J}_0(\mathrm{x})\mathrm{dx} = \mathrm{xJ}_1(\mathrm{x})$, therefore

$$Q = \frac{\pi R^2 A}{i\omega\rho}\left(1 - \frac{2\alpha i^{3/2}}{i^3 \alpha^2}\frac{J_1(\alpha i^{3/2})}{J_0(\alpha i^{3/2})}\right)e^{i\omega t} \qquad (S14)$$

To find u, it is necessary to write the flow rate and u on the same harmonic form. Assuming the flow rate is also a harmonic function and substituting into Equation (S14)

$$Q = \sum_{n=0}^{N} C_n e^{in\omega t} \qquad (S15)$$

$$\sum_{n=0}^{N} C_n e^{in\omega t} = \sum_{n=0}^{N} \frac{\pi R^2 A_n}{i\omega_n \rho}\left(1 - \frac{2\alpha_n i^{3/2}}{i^3 {\alpha_n}^2}\frac{J_1(\alpha_n i^{3/2})}{J_0(\alpha_n i^{3/2})}\right)e^{i\omega_n t} \qquad (S16)$$

After canceling out $e^{in\omega t}$, A_n becomes

$$A_n = \frac{C_n i\omega_n \rho}{\pi R^2\left(1 - \frac{2J_1\left(i^{\frac{3}{2}}\alpha_n\right)}{i^{\frac{3}{2}}\alpha_n J_0\left(i^{\frac{3}{2}}\alpha_n\right)}\right)} \qquad (S17)$$

To determine A_n, a Fourier series decomposition of the flow rate waveform should be performed to obtain Fourier coefficients (which is C_n in that case). For that purpose, Fast Fourier Transform (FFT) method can be applied to the available flow rate data. After determining C_n, $u(r,t)$can be calculated from following formula

$$u(r,t) = \sum_{n=0}^{N} \frac{C_n}{\pi R^2\left(1 - \frac{2J_1\left(i^{\frac{3}{2}}\alpha_n\right)}{i^{\frac{3}{2}}\alpha_n J_0\left(i^{\frac{3}{2}}\alpha_n\right)}\right)}\left[1 - \frac{J_0\left(\alpha_n \frac{r}{R} i^{3/2}\right)}{J_0(\alpha_n i^{3/2})}\right]e^{i\omega_n t} \qquad (S18)$$

where the term $n = 0$ corresponds to a steady pressure gradient, which is actually simple Poiseuille's flow

$$u(r,t) = \frac{2C_0}{\pi R^2}\left(1 - \left(\frac{r}{R}\right)^2\right) + \sum_{n=1}^{N} \frac{C_n}{\pi R^2\left(1 - \frac{2J_1\left(i^{\frac{3}{2}}\alpha_n\right)}{i^{\frac{3}{2}}\alpha_n J_0\left(i^{\frac{3}{2}}\alpha_n\right)}\right)}\left[1 - \frac{J_0\left(\alpha_n \frac{r}{R} i^{3/2}\right)}{J_0(\alpha_n i^{3/2})}\right]e^{i\omega_n t} \qquad (S19)$$

C_0 and Cn's are the FFT coefficients of flow waveform. The process is written on Matlab and velocity profiles are obtained. The code is given below.

[1] Womersley, J. R. Method for the Calculation of Velocity, Rate of Flow and Viscous Drag in Arteries When the Pressure Gradient Is Known. *J Physiol* **1955**, *127* (3), 553–563. https://doi.org/https://doi.org/10.1113/jphysiol.1955.sp005276.

```
% This script finds the velocity profiles inside a blood vessel for a given
% flow rate waveform. First it reads the flow rate data from a txt file and
% then finds the Fourier series coefficients by using FFT method. Finally,
% by using those coefficients, velocity profile at different time values
% are found by solving Womersley's equation.
% 04.10.2021, Burcu Ramazanlı


clear all; close all; clc;

R = 0.009;                    % Radius of vessel (m)
rho = 1000;                   % Density of working fluid (kg/m^3)
nu = 0.00000345;              % Kinematic viscosity (m^2/s)
T = 1;                        % Period of the waveform (s)

f = dlmread('wave_values.txt', '\t');
%t = f(:,1);
f_k = f(:,2);                 % Flow rate values from txt file (m^3/s)


interval= T/70;
t=0:interval:T-interval;
t=transpose(t);               % Time instants corresponding to those flow
rates


% Now that we have the flow rate waveform, convert to fft and back
% to get Fourier coefficients, need to multiply fft coefficients by 2/N
% and then divide the DC coefficient by 2

N = size(t); N = N(1);
Cn = 2*fft(f_k)/N(:,1);       % Fourier coefficients, obtained from FFT
Cn(1) = Cn(1)/2;              % DC coefficient (or, a0)

omega = 2*pi/T;
i = sqrt(-1);
ii = i^(3/2);


% Turn the signal back to time domain to see if it's correct
% Use it to check how many terms of the FT (Fourier modes) is needed to
% reproduce the waveform accurately.

waveform_f = zeros(70,1);
for n = 1:35   % number of Fourier modes to include

   % waveform_f = waveform_f + real(Cn(n)*exp(i*omega*(n-1)*t));
```

```
    waveform_f = waveform_f + real(Cn(n))*cos(2*pi*(n-1)*t/T) -
imag(Cn(n))*sin(2*pi*(n-1)*t/T);

end



figure(1); plot(t, f_k); hold on;
plot(t, waveform_f, 'r'); legend ('f_k', 'waveform_f'); grid on;


x = lin space(0,R,N);
t = linspace(0,T,N);
u = zeros (70);


% Now insert Fourier coefficients to the Womersley's velocity profile
% equation
for k = 1:N

    for y = 1:N

        r = x(y);
        u(y,k)=((2*real(Cn(1)))/(pi*(R^2)))*(1-((r/R)^2)); % Cn(1) is
actually C0

            for n = 1:35

            a_n = R*sqrt(n*omega/nu);
            J0 = besselj(0,(a_n*(r/(R))*ii));
            J0_c = besselj(0,(a_n*ii));
            J1_c = besselj(1,(a_n*ii));
            J = (1-(2/(a_n*ii))*(J1_c/J0_c));
            Kn(n) = Cn(n+1)/(pi*(R^2)*J);
            u (y,k) = u(y,k) + real(Kn(n)*(1-(J0/J0_c))*exp(i*omega*n*t(k)));

            end
        end
    end

 figure(2); plot (u, x); grid on;
```
